# Modern Methods of Sample Preparation for the Analysis of Oxylipins in Biological Samples

**DOI:** 10.3390/molecules24081639

**Published:** 2019-04-25

**Authors:** Ivan Liakh, Alicja Pakiet, Tomasz Sledzinski, Adriana Mika

**Affiliations:** 1 Department of Pharmaceutical Biochemistry, Medical University of Gdansk, Debinki 1, 80-211 Gdansk, Poland; liakh_ivan@mail.ru (I.L.); tsledz@gumed.edu.pl (T.S.); 2 Department of Environmental Analysis, Faculty of Chemistry, University of Gdansk, Wita Stwosza 63, 80-308 Gdansk, Poland; alicjapakiet@gmail.com

**Keywords:** sample preparation, oxylipins, protein precipitation, liquid–liquid extraction, solid-phase extraction, biological samples

## Abstract

Oxylipins are potent lipid mediators derived from polyunsaturated fatty acids, which play important roles in various biological processes. Being important regulators and/or markers of a wide range of normal and pathological processes, oxylipins are becoming a popular subject of research; however, the low stability and often very low concentration of oxylipins in samples are a significant challenge for authors and continuous improvement is required in both the extraction and analysis techniques. In recent years, the study of oxylipins has been directly related to the development of new technological platforms based on mass spectrometry (LC–MS/MS and gas chromatography–mass spectrometry (GC–MS)/MS), as well as the improvement in methods for the extraction of oxylipins from biological samples. In this review, we systematize and compare information on sample preparation procedures, including solid-phase extraction, liquid–liquid extraction from different biological tissues.

## 1. Introduction

Oxylipins are biologically important lipid mediators which are formed by the oxidation of polyunsaturated fatty acids (PUFAs) and include hydroperoxy, hydroxy, oxo and epoxy fatty acids [1]. Oxylipins are produced via three enzymatic pathways in a reaction catalyzed by cyclooxygenase (COX), lipoxygenase (LOX), and cytochrome P450 (CYP450) or via non-enzymatic autoxidation [1,2]. Oxylipins formed from PUFAs are octadecanoids derived from linoleic acid (18:2n-6; LA) and α-linolenic acid (18:3n-3;ALA), eicosanoids derived from dihomo-γ-linolenic acid (20:3n-6; DGLA), arachidonic acid (20:4n-6; ARA) and eicosapentaenoic acid (20:5n-3; EPA), as well as docosanoids derived from adrenic acid (22:4n-6; AdA) and docosahexaenoic acid (22:6n-3; DHA) [1]. Two of the most important long-chain PUFAs, which are precursors of oxylipins with strong anti- and pro-inflammatory properties, are EPA and ARA [3]. Most of the pro-inflammatory molecules involved in cell signaling cascades are generated from ARA, whereas the anti-inflammatory ones are derived from EPA [4,5]. Oxylipins derived from EPA are generally less potent or produced less efficiently than the analogous oxylipins derived from ARA [6,7]. ARA and EPA compete with each other for binding to COX-1 and COX-2. EPA is also an inhibitor of ARA oxidation by COX-1 (to a lesser extent by COX-2) to prostaglandin H2 (PGH2), and at the same time, ARA inhibits the conversion of EPA to prostaglandin H3 (PGH3). This close interaction between ARA and EPA modulates the production of thromboxane A2 (TXA2), thromboxane A3 (TXA3), prostaglandin I2 (PGI2), prostaglandin I3 (PGI3), PGH2, PGH3, and may have anti-inflammatory effects caused by the inhibition of the ARA metabolism [1].

**Figure 1:**
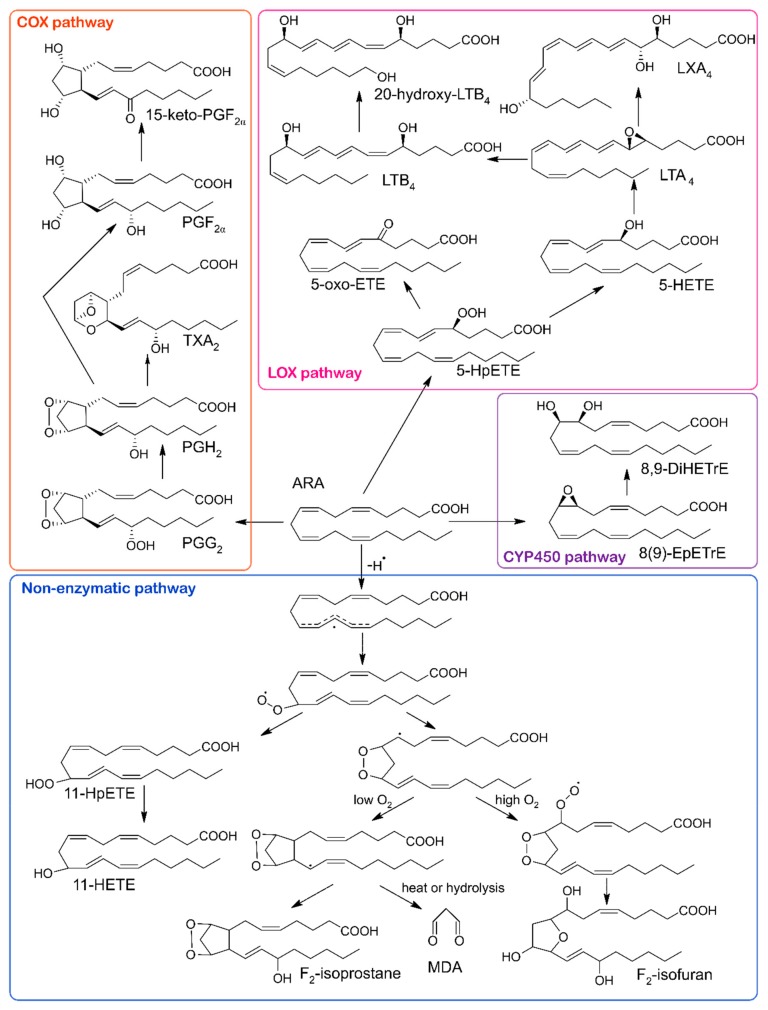
Conversion of ARA into oxylipins by COX, LOX, CYP 450 pathways and by the non-enzymatic pathway. COX, cyclooxygenase; LOX, lipoxygenase; CYP450, cytochrome P450; ARA, arachidonic acid; 5-HpETE, 5-hydroperoxyeicosatetraenoic acid; 5-oxo-ETE, 5-oxo-eicosatetraenoic acid; LTB_4_, leukotriene B4; 20-hydroxy-LTB_4_, 20-hydroxy-leukotriene B4; 5-HETE, 5-hydroxyeicosatetraenoic acid; LTA_4_, leukotriene A4; LXA_4_, lipoxin A4; 8(9)-EpETrE, 8,9-epoxyeicosatrienoic acid; 8,9-DiHETrE, 8,9-dihydroxyeicosatrienoic acid; PGG_2_, prostaglandin G2; PGH_2_, prostaglandin H2; TXA_2_, thromboxane A2; PGF_2α_, prostaglandin F2α; 15-keto-PGF_2α_, 15-keto-prostaglandin F2α; 11-HpETE, 11-hydroperoxyeicosatetraenoic acid; 11-HETE, 11-hydroxyeicosatetraenoic acid; MDA, malondialdehyde.

Eicosanoids formed from ARA are the most prevalent compounds in the oxylipin family [1]. ARA is a component of membrane phospholipids. It can be released by the phospholipase A2, as well as formed from diacylglycerol by diacylglycerol lipase [8]. ARA can be metabolized to hydroxyeicosatetraenoic acids (HETEs), dihydroxyeicosatetraenoic acids (DiHETEs), epoxyeicosatrienoic acids (EETs), prostaglandins (PGs) and thromboxane (TX), see Figure 1.

**Figure 2:**
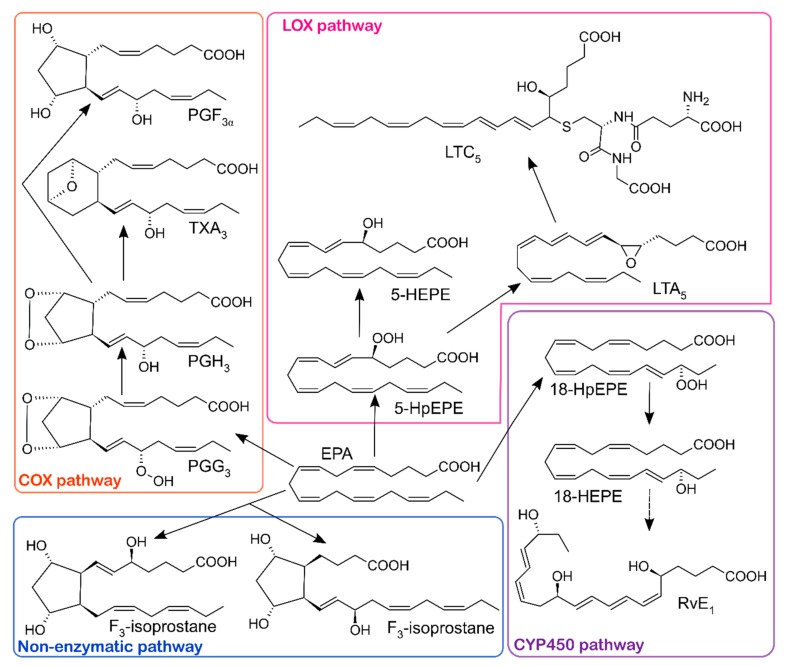
Conversion of EPA into oxylipins by COX, LOX, CYP 450 pathways and by the non-enzymatic pathway. COX, cyclooxygenase; LOX, lipoxygenase; CYP450, cytochrome P450; PGF_3α_, prostaglandin F3α; TXA_3_, thromboxane A3; PGH_3_, prostaglandin H3; PGG_3_, prostaglandin G3; LTC_5_, leukotriene C5; 5-HEPE, 5-hydroxyeicosapentaenoic acid; LTA_5_, leukotriene A5; 5-HpEPE, 5-hydroperoxyeicosatetraenoic acid; 18-HpEPE, 18-hydroperoxyeicosatetraenoic acid 18-HEPE, 18-hydroxyeicosapentaenoic acid; RvE_1_, resolvin E1.

EPA is a precursor of well-known eicosanoids, such as the PG 3-series and TXs (COX) and 5-series leukotrienes (LTs) (LOX) [9], as shown in Figure 2.

Oxylipins play a major role in regulating inflammatory processes. Depending on the precursor (n-6 or n-3 PUFA), oxylipins can respectively initiate inflammation [10,11,12] or, on the contrary, be anti-inflammatory agents [13,14]. In many tissues, oxylipins that originate from non-enzymatic autoxidation play the role of oxidative stress markers [15,16,17]. Additionally, oxylipins have a large influence on a diverse range of processes, such as ovulation, the initiation of labor, bone metabolism, nerve growth and development, wound healing, kidney function, blood-vessel tone, blood coagulation, immune responses [18,19], and play a role in pathological processes, see Table 1.

The important functions performed by oxylipins and their constant presence in biological fluids such as blood, urine, and cerebrospinal fluid (CSF), make them potential biomarkers [19]. However, the main problem in the study of oxylipins is their enormous heterogeneity associated with a large number of oxidation pathways. Depending on the type of oxidation, they form many different molecules with similar structures, chemistry and physical properties, which makes it difficult to simultaneously determine them using traditional methods [7,45,46]. In addition, most oxylipins are present at low concentrations and their detection and quantification require methods with high sensitivity [47]. Therefore, this review is aimed at describing the most popular methods of the preparation and extraction of oxylipins for quantitative analysis in various human and animal biofluids, solid tissues and cell cultures.

## 2. Sample Preparation

### 2.1. Sample Collection and Storage

Oxylipins are very unstable compounds, and this must be taken into account during the collection of materials for research. Since tissue degradation and free radical oxidation can occur within a few seconds, the material should be procured as quickly as possible: Tissue samples must be quickly frozen in liquid nitrogen, biological fluids collected and stored on ice prior to processing, and cells collected in cold solvents. Considering that non-enzymatic lipid peroxidation can occur even at −20 °C (degradation and loss of analytes have been found for some resolvins and prostanoids derived from DHA and EPA), all samples should be stored at −80 °C, and freeze/thaw cycles should be avoided [48]. To prevent this problem, some antioxidants can be used, and this is described below in Section 2.2. Golovko et al. observed that the storage of brain tissue powder at −80 °C for about four weeks resulted in a two- to four-fold decrease in PG mass [49]. Even the short storage of blood at room temperature before further processing has a huge impact on the concentration of several oxylipins in the plasma: After the storage of whole blood for 60 min, the levels of several oxylipins are greatly reduced (e.g., 15-HETE and 14(15)-EpETrE), whereas other analytes are formed ex vivo (e.g., PGE2) [50]. Leaving samples in the centrifuge for several minutes after centrifuging and prior to collection and freezing, can also lead to a significant decrease in the levels of some oxylipins [50].

When using clinical material, attention should be paid to such a factor as the use of heparin in the treatment of patients, because it leads to heparin-induced phospholipase A2 activity and the elevation of oxylipin levels. In addition, it is advisable to collect the samples at the same time during the day, in order to reduce the potential impact of a circadian rhythm, which can affect several oxylipins, whose concentrations decrease during the day after the morning peak [51]. In serum, coagulation is, in part, mediated by the ARA cascade and causes a massive (ex vivo) formation of several oxylipins (TxB2, 12-HETE), also the detectability in the serum of low-concentration mediators (e.g., resolvins) is higher compared to plasma [50].

In animal experiments, it is also necessary to consider the method used for the euthanasia of the animal and the subsequent processing of the brain sample. It was shown that during decapitation, the level of PGs in the brain of rats was 10–40 times higher than that of rodents euthanized by focused microwave radiation. This difference is primarily the result of the thermal inactivation of enzymes involved in the post-mortem formation of PGs, and to a lesser extent, the capture or destruction of PGs under the action of microwave radiation [49]. Microwave irradiation at a temperature of 70–80 °C prevents postmortem induction in brain eicosanoids and allows the measurement of true levels of eicosanoids, while problems such as significant PG heat-destruction or the trapping of denatured proteins were not detected [49]. In addition, in order to prevent further PG formation after death, the proteins may be heat-denatured in a boiling water bath for 5 min before analysis [52]. 

Another problem associated with container transfer loss occurs when using, as a surrogate matrix, phosphate buffered saline (PBS)–methanol (MeOH) containing butylated hydroxytoluene (BHT)/ethylenediaminetetraacetic acid (EDTA) (called PMC). Due to the lipophilic nature of the oxylipins and the lack of protein in the PMC matrix, there may be nonspecific binding, which leads to the loss of analytes due to adsorption, especially to the hydrophobic surface of polypropylene materials during transfer with test tubes, bubbles and tips in sample preparation. Usually, these losses during sample preparation are compensated for by the calibration curve; however, with initially low levels of oxylipins in the sample and/or a long transfer time, there can be a significant loss of analytes [53].

Various undesirable components may be present in blood collection tubes available on the market. Silicones can be used as stoppers lubricants or as internal surface coatings. To control the surface wetting density and viscosity surfactants (polyethylene glycols or polyvinylpyrrolidones) and polymeric gels are used. In addition, coagulation activators/inhibitors, polymers and plasticizers presented on the plastic tube walls and rubber stoppers can be added to this list. All the described agents released from plastic containers can be detected after matrix-assisted laser desorption/ionization time-of-flight (MALDI TOF) mass spectrometry and disturb the analysis results. [54]. Furthermore, some substances such as ultraviolet (UV) stabilizers from standard polyvinyl chloride tubes or plasticizers from standard polypropylene microcentrifuge tubes can interfere with liquid chromatography–mass spectrometry (LC–MS) applications [55,56] and analysis by gas chromatography–mass spectrometry (GC–MS) [57]. Nevertheless, in the case of collecting urine samples, polypropylene tubes are used throughout the subsequent process to avoid the binding of eicosanoids to glass surfaces [58].

### 2.2. Pre-Extraction Additives

#### 2.2.1. Antioxidants

One of the problems that reduce the accuracy of the analysis of biological samples is the formation of oxylipins after sampling. To prevent this process from occurring, COX and soluble epoxide hydrolase (sEH) inhibitors can be used. For example, it is recommended to add 100 mM trans-4-[4-(3-adamantan-1-yl-ureido)cyclohexyloxy]-benzoic acid (t-AUCB) to inhibit the soluble epoxide hydrolase in human plasma. In addition, esterase and protease inhibitors are added to the samples to prevent enzymatic degradation or the formation of oxylipins. [59]. To prevent the formation of ex vivo eicosanoids in urine samples, indomethacin should be added to them immediately [58,60,61]. Furthermore, to prevent the oxidation and breakdown of oxylipins, plasma samples can be stored in MeOH containing Paraoxon—an acetylcholinesterase inhibitor, 12-(3-adamantan-1-yl-ureido)dodecanoic acid (AUDA)—an inhibitor of sEH, and phenylmethylsulfonyl fluoride (PMSF)—a serine protease inhibitor of thrombin [62,63]. 

Triphenylphosphine (TPP) and radical-scavenging BHT can be added to the tissues during sample collection [47,64]. TPP was used to reduce peroxides to their monoatomic equivalents, and BHT was used to quench radical-catalyzed reactions. Both reagents prevent the conversion of PUFAs to peroxyl radicals [47], and oxylipin degradation or formation (e.g., 11-HETE, 9-HETE, isoprostanes (IsoP)) by autoxidation during sample preparation [50]. In the analysis of lipids, different concentrations of BHT (0.005% to 0.2%) can be used [10,47,65,66]. To assess the need for antioxidants, Golovko et al. analyzed three identical brain samples with 0.1% BHT, 0.005% BHT or without BHT added to the acetone and chloroform used in the extraction. It was found that using only 0.005% BHT prevents the reduction of the mass of 6-oxo-PGF_1_, whereas 0.1% BHT produces a precipitate in brain samples, which can clog the LC system [49].

Considering the ability of hydroxylated lipids to be converted to glucuronides and other conjugates prior to isolation, Newman et al. incubated urine samples for 3 h at 37 °C with 400 units of Helix pomatia type H-1 glucuronidase to release dihydroxy lipids from their glucuronides [67]. Morgan et al., during the procedure of the synthesis of internal standards, used an addition of 10 μL of SnCl_2_ (100 mM in water) per mL of sample before extracting lipids from samples with immune cells, for 10 min at room temperature before the extraction to reduce more hydroperoxides unstable to alcohols [68].

#### 2.2.2. Standards

In order to normalize the extraction efficiency and instrument response, the internal standard (IS) is added to the sample before extraction. Deuterated ISs play an important role in the extraction and storage process. Since the deuterated IS is either a similar lipid metabolite or a molecule with similar chemical characteristics, the IS will have the same extraction efficiency and decomposition rate as a lipid metabolite, which will allow the calculation of the amount that is lost as a result of the extraction process or degradation [69]. For oxylipin analysis by mass spectrometry (MS), a mixture of deuterated species is used, in addition to a sufficient number of deuterium atoms (^2^H), and analytes can be labeled with ^13^C atoms [70]. Because it is impractical to use an IS for each species analyzed, at least one IS for each lipid class is used, and is selected based on structural similarities [71]. Wang et al. used only 26 deuterated ISs for the analysis of 184 eicosanoids by ultra-high-performance liquid chromatography (UHPLC)/MS [72]. The most commonly used ISs are d4-PGE_2,_ d4-PGD_2,_ d8-12(*S*)-HETE, d8-5(*S*)-HETE, d4-PGF_2α,_ d4-LTB_4,_ d11-14,15-DiHETrE, d4-9(*S*)-HODE, d4-12(13)-EpOME, d11-14,15-EET, d11-8,9-EET and d11-11,12-EET [39,73,74,75,76]. Although it is important to choose ISs that are not altered during the extraction, Hennebelle et al. found that d4-PGE_2_ used as the IS underwent degradation during the plasma preparation (hydrolysis process), and, as a consequence, oxylipins that were analyzed with d4-PGE_2_ cannot be quantified. These included THF-diol, epoxy-keto-octadecenoic acid (EKODE), PGE_1_, PGD_1_, PGF_2α_, PGE_2_, PGD_2_, PGJ_2_, PGB_2_, PGE_3_, PGD_3_, 15-deoxy-PGJ_2_, resolvin E_1_, 9,12,13-TriHOME, 9,10,13-TriHOME and 11,12-,15-TriHETrE [64]. 

However, according to the variability of preparation procedures, the use of only one IS is not enough to overcome that problem, and several deuterated ISs can be used [51,63,77]. For example, Yang et al. used deuterated ISs for the extraction of prostaglandins, diols, epoxides and other oxylipins. ISs were added to the samples before extraction (d4-6-keto-PGF_1α_, d4-PGE_2_, 10,11 DHHep, d6-20-HET-, d4-9-(S)-HODE, d8-5-HETE, d8-11,12-EET). After that, to calculate the recovery rates of each IS, another standard synthetic acid, 1-cyclohexyluriedo-3-dodecanoic acid (CUDA), was added before analysis [47]; 1-phenylurea-3-hexanoic acid (PUHA) may also be added with CUDA as a quality marker for the analysis [73].

### 2.3. Extraction Methods

It is not always possible to analyze a sample without first isolating the components from the natural matrix. At the same time, in order to reduce the lower limit of detection and increase the sensitivity of determinations, as a rule, it becomes necessary to concentrate them with respect to the matrix components present in the tissue under study. In this case, the separation procedures can significantly simplify the analysis and increase its selectivity, eliminating the influence of interfering impurities. 

Considering that oxylipins are present in very low concentrations and that many species are very unstable at room temperature, sample preparation and extraction should be carried out in cold conditions [49,78,79]. Some oxylipins may also be formed during the extraction process. The homogenization stage may activate the synthesis of eicosanoids [49,80]. Due to the high activity of the nonenzymatic and enzymatic processes in blood, urine, solid tissues, or other samples from humans and animals, they often contain very few intact PGs; therefore, sometimes measuring PG metabolite levels is more important [58]. Similarly, more meaningful results might be expected from the determination of oxidized plasma prostanoid metabolites, which are assumed not to be formed as rapidly during the sampling procedure compared to peripheral plasma prostanoid concentrations [81].

Sample preparation includes steps such as adding solvents, acids and antioxidants; extraction; homogenization; centrifugation; the hydrolysis of esterified lipids; the derivatization process. In this review, we will focus on the most commonly used extraction methods that are implemented in the determination of oxylipins.

#### 2.3.1. Protein Precipitation

The extraction of free oxylipins from a biological matrix, such as plasma or tissue, is difficult. Analytes have a wide polarity range and are prone to decomposition during auto-oxidation (all oxylipins) and when treated with a base (PG) or acid (epoxy-FA). If analyte concentrations significantly exceed the limit of quantitation (LOQ) of the instrument, the sample can be directly injected after protein precipitation (PPT) by dilution with organic solvents. However, most analyses require the pre-concentration of the samples using liquid–liquid extraction (LLE) or, most often, solid-phase extraction (SPE); however, in almost all cases, prior PPT is required [50]. In addition to removing the protein, adding an organic solvent to a biological fluid also disrupts the bonds between the metabolites and the proteins present in the solution. As a result, the obtained concentrations of metabolites are total metabolite concentrations equivalent to the sum of the bound and unbound (free) metabolite concentrations [82]. Additionally, PPT with acids can catalyze the hydrolysis of certain conjugates, such as glucuronides and sulfates [83]. 

Satomi et al. compared PPT using different water-soluble organic solvents such as MeOH, ethanol (EtOH), isopropanol (IPA) or acetonitrile (ACN), followed by methyl tert-butyl ether (MTBE)-based lipid extraction, to determine which would be the best method for sample preparation for LC–MS-based lipidomics analysis. ACN deproteinization is less effective than alcohol, potentially due to insufficient protein denaturation, which causes lipid decomposition through lipase activation. Therefore, protein precipitation by alcohol was evaluated as the best lipid extraction method [84]. MeOH extraction was appropriate for partly hydrophilic lipid species, and IPA was effective for hydrophobic lipids such as triacylglycerols. The best approach to cover a wide range of lipid species using a simple preparation procedure, in practice, is thought to be EtOH extraction [84]. 

Similar to lipid extraction, when using PPT for the extraction of oxylipins, various types of solvents can be used, their choice of which depends on the type of tissue and the class of the analyte to be determined [39]. PPT by adding two volumes of water-miscible organic solvents, such as MeOH, generally provides a high extraction efficiency (>90%) [85]. However, in the case of the analysis of eicosanoids, this extraction method is not very suitable due to the presence of eicosanoids in the samples at very low concentrations, and precipitation together with the proteins to which they bind unspecifically [85]. Lee et al. used methanol-based protein precipitation, which was followed by the LC–tandem mass spectrometry (MS/MS) analysis of 20 oxylipin levels in the serum of women with endometriosis [71]. Heemskerk et al. performed PPT by the addition of methanol to an adipocyte-conditioned medium or plasma to analyze adipose tissue PUFA synthesis and anti-inflammatory lipid and oxylipin plasma profiles using LC–MS/MS [86]. Although, according to Satomi [84], alcohol precipitation is superior to that of ACN, other authors obtained good results using ACN [87]. Chocholoušková et al. applied ACN for the denaturation during the UHPLC/MS determination of oxylipins in human plasma and found a high efficiency of ACN in protein precipitation [39]. Zein et al. used ACN in PPT for the HPLC-electrospray ionization (ESI)-MS/MS analysis of free fatty acids, eicosanoids and docosanoids in human gingival crevicular fluid (GCF), saliva and serum [88]. In addition, Wang et al. used ACN PPT for the analysis of free arachidonic acid in plasma with the use of LC–MS/MS [89].

For the most efficient protein removal, organic solvents such as MeOH and ACN can be combined with zinc sulfate [90]. Kortz et al. used PPT with a solution consisting of MeOH:zinc sulfate (4:1 *v*/*v*) before performing an online SPE-LC-MS/MS analysis of PUFAs and eicosanoids in human plasma [91]. Additionally, Klawitter et al., to measure concentrations of 15-F2t-isoprostane in human plasma and urine samples, used PPT with MeOH/zinc sulfate. Afterwards, the PPT samples were injected into the HPLC system and extracted online. No carry-over and no matrix inferences such as ion suppression or enhancement were observed [92]. Good results can also be achieved with serial PPT using different solvents. Bessonneau et al. compared the performance of the non-lethal in vivo solid-phase microextraction (SPME) sampling method for rat plasma with PPT (acetone/hexane/chloroform) for monitoring the time profile of blood eicosanoids. The results obtained for 12-HETE and ARA were significantly correlated with those obtained using conventional PPT [93].

#### 2.3.2. Liquid–Liquid Extraction

Liquid–liquid extraction (LLE) is a widely used sample preparation method for extracting all major classes of lipids, including phospholipids, ceramides, sphingomyelins, and cholesterol esters [94]. Until recently, the chloroform:MeOH Folch [95] and Bligh and Dyer [96] methods of extraction were the most common methods. In the Folch procedure, a 2:1 chloroform:MeOH mixture is added in a volume 20 times higher than that of the sample. The subsequent addition of saline solution allows a lower layer to be obtained consisting of all lipids, and an upper layer consisting of contaminants. At the final stage, a solution consisting of a chloroform:MeOH:water in a ratio of 8:4:3 is used to affirm lipid separation in the chloroform layer [95]. The adapted procedure of Bligh and Dyer differs in the amount of solvent used (3 mL of MeOH:chloroform mixture 2:1 per gram or mL of the sample). After stirring, 1 mL of chloroform and 1.8 mL of water were added to separate the solution into two phases. A shorter extraction time and the use of chloroform to re-extract tissue improves lipid yields compared to the Folch procedure [97]. However, the main disadvantage of the Folch et al. or Bligh and Dyer protocols is that they lead to the quantitative extraction of most lipids, including those that can be present in very high concentrations in biological tissues (cholesterol, triacylglycerols and phospholipids). High levels of these lipids can interfere with the analysis of oxylipins, which are usually present in very low concentrations [72]. Using the chloroform–MeOH or chloroform–methanol–water method, both hydrophilic and lipophilic substances can be extracted simultaneously; however, because using chloroform is problematic in LC–MS, such an approach requires the further removal of chloroform by lyophilization [82]. Another approach for the extraction of the main classes of lipids is separation using MTBE [98]. A comparison of the extraction efficiency of classes of lipids in human plasma by the MeOH, MeOH:EtOH (1:1) and MTBE methods shows the best result for the separation of lipids using MTBE (3,125 metabolites were detected using LC–TOF with C18 and HILIC columns in positive mode summary) [98]. There is little information about the use of MTBE for the extraction of oxylipins, but it is known that Rund et al. successfully used MTBE extraction for the analysis of IsoP and IsoF formed in HCT116 cells [59].

##### Biofluids

When determining oxylipins in plasma, the ratio of plasma to precipitant is between 1:1.35 *v*/*v* to 1:4 *v*/*v* in various studies. The nature of the extraction solvent has a profound effect on the process extraction efficiency [82]. Various types of organic solvents can be used to extract oxylipins. In the Fleming Laboratory (Frankfurt, Germany), double extraction with ethyl acetate (EA) is used to determine levels of fatty acid epoxides in murine plasma or bone marrow extracellular fluid, obtained from flushed-out femurs. In addition, oxylipins were extracted from plasma with sodium acetate, followed by extraction with EA [99]. Using a modification of the Golovko acetone extraction method [49], Pier et al. identified 10 different PGs in human ovarian follicular fluid [100]. For the determination of seven F2-isoP isomers among classes III, IV, and VI in the blood plasma of pregnant women, Larose et al. developed a method including hydrolysis by KOH, double pre-extraction with hexane, and consequent triple extraction with EA:hexane (3:1) [101]. Hall and Murphy used extraction by the Bligh and Dyer method, substituting methylene chloride for chloroform to quantitate production of 5-HETE, 5-HPETE, 5-oxo-ETE in red blood cell (RBC) ghosts [102]. 

##### Solid Tissues

Unlike biofluids, the extraction of oxylipins from solid tissues is preceded by a homogenization process, which itself can activate the synthesis of some oxylipins. To prevent this, special additives may be used, see Section 2.2 [79]. 

The most common LLE method for tissue extraction involving chloroform is the Bligh and Dyer method [78,94]. However, due to the wide range of extracted lipids, matrix effects and the response of analytical equipment, the authors also used other solvents for LLE from tissue. In order to increase the extraction of eicosanoids, reduce chemical background noise and reduce the preparation time, Brose et al. changed the LLE protocol by replacing acetone:chloroform with MeOH. Using a smaller volume of solvents, modified single-stage extraction with MeOH resulted in a much higher (96.7 ± 9.9%) extraction of the internal standard, which may be the result of eliminating analyte loss through transfer/evaporation steps [52]. In another work, Brose et al. used LLE with acetone to extract prostaglandins and isoprostanes (PGE2, PGD2, isoPGE2 such as PGE2, entPGE2, 8-isoPGE2, 11β-PGE2, PGD2, and 15(R)-PGD2) from murine brain [103]. Urban et al. established that for the extraction of PGs from pig brain tissue, the use of an EtOH:10 mM phosphate buffer (85:15) as the extraction solvent, showed better results compared to EtOH:dichloromethane (1:1), MeOH:10 mM phosphate buffer (85:15) and 10 mM phosphate buffer [104].

##### Cell Cultures

LLE with hexane:EA is very often used for studying endogenous oxylipins from cell cultures. Yang et al. used this method to investigate levels of PGD2, 15-keto-PGE2, 13,14-dihydro-15-keto-PGE2, PGD3, 8-iso-PGE2, 8-iso 15-keto PGF2α, PGF3 α, and 8-iso PGF3 α in human non-small-cell lung cancer cells (A549) and human colon carcinoma cells (DLD-1) [66]. Kempen et al. used hexane:EA (1:1, *v*/*v*) LLE for the quantification of ARA metabolites (PGE2, 11-HETE, 5-HETE,12-HETE) from human lung cancer cells H1299 and A549, and a rat leukemia cell line RBL-1 [105]. In addition, using hexane:EA (1:1, *v*/*v*) LLE, Schroeder et al. analyzed endogenous levels of eicosanoids (PGE2, 5-HETE, 12-HETE, 15-HETE, and 13-HODE) in cell lysates of squamous cell carcinoma cell lines of the head and neck (HNSCC) [106]. Morgan et al. used the hexane:IPA:acetic acid (HAc) LLE procedure to measure esterified oxylipins generated by immune cells: hydro(pero)xyeicosatetraenoic acids (H(p)ETEs), hydroxyoctadecadienoic acids (HODEs), hydroxydocosahexaenoic acids (HDOHEs) and keto-eicosatetraenoic acids (KETEs), attached to either phosphatidylethanolamine (PE) or phosphatidylcholine (PC). Using this extraction method allows the simultaneous monitoring of up to 23 different oxylipins, with better recoveries of standards and analytes than the classical Bligh and Dyer method [68]. Michaelis et al. used LLE with hexane/EA (1:1, *v*/*v*) to determine cytochrome P450 (CYP) epoxygenase-derived epoxyeicosatrienoic acids (EETs) in bovine endothelial cells [107]. PGD2 and PGE2 were measured by Cao et al. in culture supernatants from A549 cells and RAW 264.7 cells after hexane/EA (1:1, *v*/*v*) extraction [108]. All extraction steps must be performed under conditions with minimal light levels to reduce the potential for the photodegradation of the eicosanoid metabolites [106]. 

#### 2.3.3. Solid-Phase Extraction

**Figure 3:**
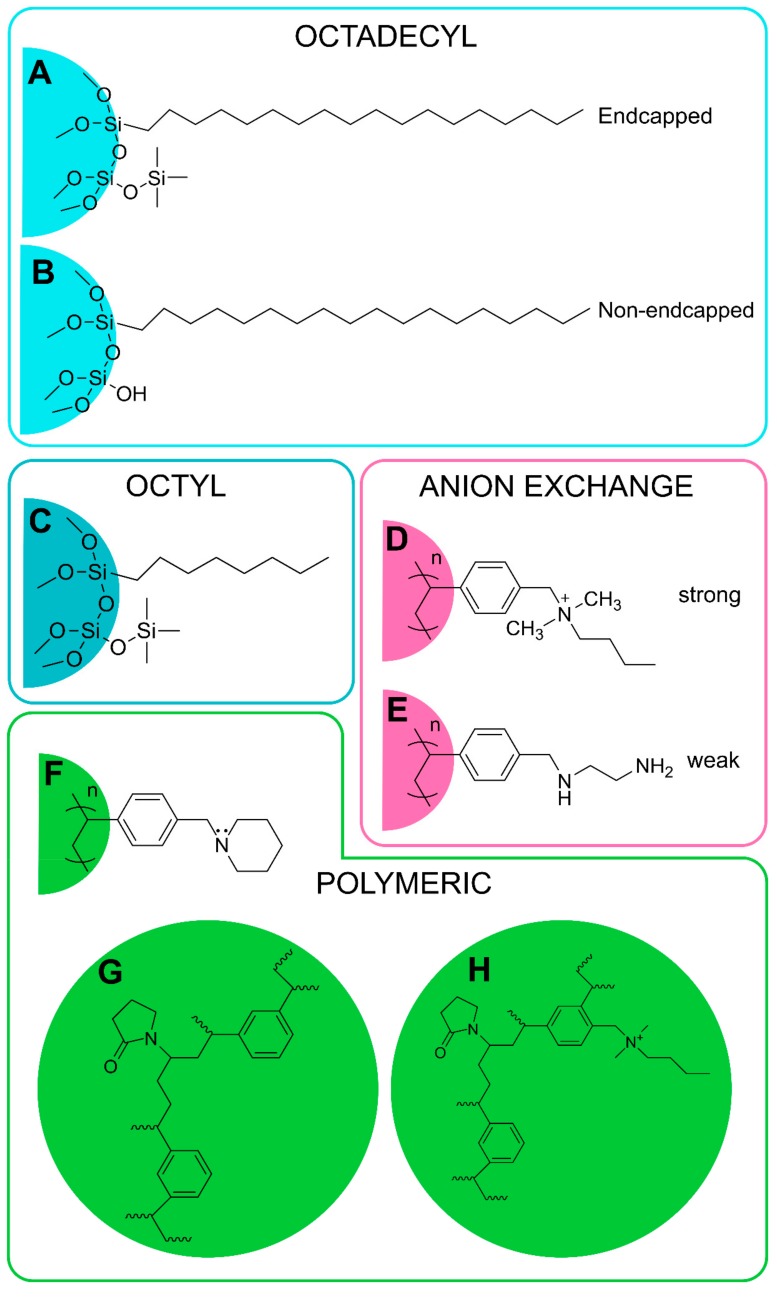
Examples of the most commonly used types of solid-phase extraction (SPE) phases for oxylipin extraction. A: Strata C18-E; B: Chromabond C18; C: Discovery DSC-8; D: Strata-X-A; E: Strata-X-AW; F: Strata-X; G: Oasis HLB; H: Oasis MAX.

The basis of the SPE method is the selective separation of analytes between the liquid and the solid phase. The main goal is to remove the compounds that cause matrix effects during the analysis and to concentrate analytes, thus increasing the sensitivity as well as improving the detection limits. By removing interfering compounds and impurities, SPE thereby protects analytical systems and increases efficiency, and when proper solvents are used for elution, tunable selectivity becomes possible [97]. The most commonly used SPE cartridges may be reversed phase (RP) (C18), normal phase (silica) and ion exchange (anion or cation) phase. The basic principle for the use of reverse-phase SPE is that aliphatic fragments in oxylipins can interact with non-polar stationary phases. Silica retains polar compounds, typically used for sample clean-up. Anion-exchange polymer-based resins selectively retain oxylipins based on both hydrophobic and anion-exchange interactions. Polymeric sorbent (containing both lipophilic and hydrophilic functional groups) allows the retention of more lipid metabolites [109], see Figure 3. 

Usually, the process consists of several stages. At the first stage, the cartridge containing the solid sorbent is conditioned with a suitable solvent. Then, a tested sample is loaded onto it and subsequently, analytes bind to the sorbent. In the next step, the cartridge is washed to remove unwanted impurities. At the last stage, the analytes are eluted from the cartridge with a solvent, which is selected taking into account the chemical and physical characteristics with respect to the analyte [110].

##### Biofluids

###### Blood/Serum/Plasma

Serum and plasma samples can be directly loaded onto the SPE cartridge [51,76]; however, they usually require pretreatment, since analytes can be bound to proteins, which reduces SPE extraction. To break the bonds between the analyte and the protein, one of the following methods can be used: Changing the pH of the sample to the extreme (pH < 3 or pH > 9) with acids or bases in the concentration range of 0.1 M or more, the above-described protein precipitation using polar solvents (ACN, MeOH or acetone), and treatment with acids or inorganic salts (formic acid (FAc), perchloric acid, trichloroacetic acid, ammonium sulfate, sodium sulfate or zinc sulfate). Less commonly, ultrasonic treatment of biological fluid for 15 min is used [109]. The calcium chelator, EDTA, which can sequester calcium ions and then inhibit phospholipase A2, is used to prevent the ex vivo formation of eicosanoids during the preparation of plasma samples [78]. Oxylipins can either be bound to circulating plasma proteins, such as albumin or can be included in lipoproteins, and may be released during the elution step (e.g., EETs) [111]. This is due to the dissociation of fatty acids from lipoproteins or albumin as a result of the denaturation of proteins by MeOH as an eluent of SPE. It can lead to an overestimation of oxylipin levels, so it is very important to use a sufficient number of IS during the extraction [72]. 

Considering the fact that there are a wide variety of sample preparation methods for the efficient pre-concentration and extraction of oxylipins from the matrix, Ostermann et al. compared SPE protocols on various cartridges for the analysis of free (non-esterified) oxylipins in human plasma [112]. Classical reverse-phase (RP) material was compared to novel polymeric stationary phases with polar groups, such as Strata X (Phenomenex, Torrance, CA, USA) or Oasis HLB (Waters, Eschborn, Germany). For the extraction of weakly acidic oxylipins from biological samples, materials with anion-exchange properties were used. Compared with other described sample preparation methods, the SepPak tC18 SPE protocol from Serhan lab (Boston, MA, USA [113]) and SPE column (6 mL, 500 mg, 37–55 μm; Waters, Boston, MA, USA), most effectively extracted free oxylipins from plasma, and for many analytes (especially for non-polar epoxides), this protocol gave the highest peak area [112]. 

However, the extraction efficiency depends not only on the type of column used but also on the SPE conditions (solvents used for column conditioning, washing and elution). Galvão et al. compared their own method of extracting eicosanoids from plasma samples by SPE using Sep-Pak C18 cartridges (500 mg, 2.8 mL) with two previously published methods, in which EtOH, hexane and EA were used for washing and elution. The selection criteria were: A lower consumption of organic solvents and a greater recovery of eicosanoids. Using 2 mL MeOH, 2 mL water/0.1% HAc for column conditioning, 2 mL water/0.1% HAc for the removal of impurities and 2 mL MeOH/0.1% HAc for sample elution, they achieved a better recovery compared with other methods, especially for the extraction of LTs, HETEs, and lipoxin (LX) A4 [114].

###### Urine

Before target extraction, urine samples can be diluted with a buffer with an appropriate pH or with water. [115,116]. To better dissolve compounds from urine, acid or base hydrolysis is used for basic and acidic compounds, respectively. The urine is heated for 15–20 min after adding a strong base (for example, 10 M KOH) or an acid (usually concentrated HCL), then cooled and diluted with the buffer, and the pH is adjusted accordingly for the SPE procedure. Enzymatic hydrolysis can also be used [109]. For extraction, organic acids are suggested, since mineral acids promote the faster dehydration of urine PGE2 to PGA2 [58].

Sterz et al. compared SPE methods when developing a method for the quantitative determination of seven types of eicosanoids in urine. Each tested SPE cartridge allowed the extraction of all types of eicosanoids (except 12-HETE). C18 RP-SPE (Bond Elut C18) was ideal for extracting LTE4 and 12-HETE. Polymeric SPE (Oasis^®^HLB, Waters, Eschborn, Germany and Strata X33u, Phenomenex, Aschaffenburg, Germany) was favorable for the extraction of 2,3-dinor-TxB2 and 11-dehydro-TXB [117]. Polymeric RP/strong anion exchange SPE (Oasis^®^MAX, Waters, Eschborn, Germany) was good for most analytes (optimal for extracting PGG2α species and tetranor PGE-M). Polymeric RP/weak anion exchange SPE (Easy cartridge) showed the worst results for the extraction of eicosanoids compared to all the others [117]. Medina et al. carried out a study of three types of different SPE cartridges: Strata X-AW (100 mg, 3 mL; Phenomenex, Torrance, CA, USA), C18 Sep-Pak classic cartridge, and OasisHLB (both 200 mg, 6 mL; Waters, Milford, MA, USA), in order to establish the most efficient one for the extraction of 13 eicosanoids in human urine [118]. The best recovery was shown for Strata X-AW cartridges (93–107%), when the recovery for Oasis HLB and C18 Sep-Pak cartridges was between 59–71%. Due to the weak ion-exchange interaction of the resin, Strata X-AW provided better reproducibility while the other types of cartridges showed unrepeatable extraction, which depended on the IsoPs nature [118].

###### Other Biofluids

Practically any biological fluid from which it is necessary to extract oxylipins can be subjected to SPE. Gouveia-Figueira et al. used Waters Oasis HLB SPE cartridges (60 mg, 30 μm), with the elution of 3 mL ACN, 2 mL MeOH, and 1 mL EA to isolate oxylipins in bronchial wash and bronchoalveolar lavage samples [119]. Panthi et al., to develop an optimal method for isolating lipid mediators of inflammation in the tear film, compared different PPT and LLE techniques with one SPE method. Only in the case of SPE (Strata X-AW, 33µ polymeric weak anion; Phenomenex, Torrance, CA), it was possible to isolate six analytes of PG and IsoP in small volumes of tears [120]. Using SPE (Sep-Pak C18, 500 mg, 6 mL; Waters, Boston, MA, USA), Giera et al. extracted several important lipid mediators in human synovial fluid [121]. SPE cartridges (HyperSepRetain PEP; Thermo Fisher Scientific, Waltham, MA, USA) were also useful to exclude impurities in the case of the preparation of platelet-rich plasma samples [122]. Wang et al. used SPE (C18 column, 500 mg; Biotage, Uppsala, Sweden) to isolate oxylipins generated by phagocytes from blood plasma [123].

When examining the levels of oxylipins in human milk samples, the pre-extraction procedure is not necessary. For sample preparation for SPE, only double centrifugation is required to remove the fat layer [124]. Robinson et al. used SPE to determine oxylipin levels in human milk. Using Oasis HLB 96-well plates for SPE (Waters), they quantified eighteen oxylipins: 6-keto- PGF1α, TXB2, PGE2, LXB4, LXA4, LTB4, 15-hydroxyeicosatetraenoic acid (HETE), 12-HETE, 5-HETE, resolvin (Rv) D1, RvD2, 7(S)-maresin (MAR) 1,7(R)-MAR1, protectin D1, protectin DX, 18-hydroxyeicosapentaenoic acid (HEPE), 14-hydroxydo-cosahexaenoic acid (HDHA), 17-HDHA [125]. For the same purposes, Wu et al. used Oasis HLB cartridges (60 mg, 30 µm; Waters, Milford, MA, USA), which allowed the extraction and simultaneous detection of 31 oxylipins from human milk [124]. Thus, SPE is a popular method for extracting and concentrating oxylipins from various biological fluids due to the high efficiency it displays with respect to removing impurities from the sample.

##### Solid Tissues

Solid tissues also need some pretreatment. They are homogenized either in water, in a polar organic solvent (e.g., MeOH or ACN), or in mixtures of water with these solvents, for RP or ion exchange cleanup procedures. Together with the iron chelator EDTA, diethylenetriaminepentaacetic acid (DTPA) may be included in the extraction process before homogenization to limit the formation of eicosanoids [79]. Care must be taken with the choice of pH for extraction. Despite the fact that acidic conditions stabilize the free carboxylic acid form of eicosanoids, and reduce protein binding [126], eicosanoids can be altered by the extraction procedure at extreme pH values [127]. Tissue extracts obtained with mid-polar to non-polar solvents can be processed using normal-phase SPE procedures. After centrifugation or filtration to remove the precipitated proteins and solids, the pH of the sample may need to be adjusted. The analyte may adsorb onto the SPE packing or alternatively, it may simply pass through, free from interferences [109]. 

Among solid tissues, oxylipin levels are most often examined in brain tissue that has high moisture and fat contents. This must be taken into account when choosing the extraction solvent for such lipophilic oxylipins as eicosanoids because they will bind to fats. Solvents for the SPE of eicosanoids from brain tissue must dissolve the eicosanoids, permeate the matrix of the brain tissue, destroy the tissues, release the eicosanoids and, finally, cause protein precipitation [126]. For the extraction of oxylipins from rat brain, different authors successfully used various methods and types of cartridges: Arnold et al. used Bond Elut Certify II columns to identity EETs in rat brain [9]; Masoodi et al. used C18-E columns (500 mg, 6 mL; Phenomenex, Macclesfield, UK) for the analysis of LTs, resolvins, protectins and related hydroxy-fatty acids in rat brain [113]; Yue et al. used Oasis^®^HLB (1 cm^3^, 30 mg, 30 µm; Waters Corporation, Milford, MA, USA) for the determination of bioactive eicosanoids [126]. In addition, Yue et al. found that MeOH, EtOH or acetone, together with phosphoric acid or FAc, have a similar extraction efficiency, but MeOH and FAc evaporate more easily and are more compatible with SPE (Oasis^®^HLB, 1 cm^3^, 30 mg, 30 µm; Waters), and anhydrous ACN is a stronger mobile phase than MeOH and more efficiently elutes eicosanoids [126]. 

Blewett et al. used SPE (Oasis SPE cartridge; Waters, Milford, MA) with the HPLC–ESI–MS method for the simultaneous determination of 23 eicosanoids in rat kidney tissue [128]. Le Faouder et al. optimized the sample preparation and the extraction process with an extraction yield of 80%, ranging from 65% to 98%. Using SPE on a C18 cartridge (15 mL, 200 mg; Macherey Nagel) they obtained the separation of 26 PUFA derivatives in colonic tissues of mice [129]. Weylandt et al. used SPE with an anion exchange column (Bond Elut Certify II; Agilent, Santa Clara, CA, USA) to determine the profile of lipid mediators formed from PUFAs (18-HEPE, 17-HDHA,15-HETE) in mouse liver [130]. Jelińska et al. successfully used SPE cartridges (Bakerbond C18, 3 mL, 500 mg, from J.T. Baker. Hampton, NH, USA) to extract eicosanoids (13-HODE, 9-HODE, 15-HETE, 12-HETE, 5-HETE) and PGE2 from 7,12-dimethylbenzanthracene (DMBA)-induced tumors in rats for further LC–MS/MS analysis [131].

##### Cell Cultures

Cell culture media also require the dilution of the media with water or a buffer at the proper pH to ensure that the analyte is freely dissolved in the sample. If a particulate-laden cell culture medium is difficult to pass through the SPE device, it may need to be vortexed and centrifuged prior to SPE [109]. The direct analysis of oxylipins from the culture medium is complicated by the fact that they can be rapidly metabolized in vitro (e.g., PGE2 and PGD2) [58].

SPE has been widely used for the analysis of oxylipins in cell culture supernatants and lysates and showed greater efficiency both in the number of analytes found and in the values of recovery, lower limits of detection (LLOD) and lower limits of quantitation (LLOQ) [2,85] compared to LLE. Deems et al. developed a procedure for isolating eicosanoids from media and cells from a cell culture. After the medium collection, the eicosanoids were isolated from medium by SPE using Strata-X SPE columns (Phenomenex, Torrance, CA, USA), which allowed over 60 discrete chemical species of eicosanoid to be identified [132]. Wang and DuBois used 6 mL Sep-Pak C18 cartridges (Waters Associates) or a 6 mL octadecyl silica (ODS) column to extract eicosanoids from the cell-free culture medium. More polar materials were removed by subsequent elution with 15% aqueous EtOH. After extraction, the recoveries of 6-keto-PGF1α, TXB2, PGE2, PGF2α, LTB4, LTC4, 5-HETE, 12-HETE, and 15-HETE were 90.5%, 90.6%, 92.5%, 98.1%, 86.1%, 98.3%, 95.3%, 99.8%, and 92.8%, respectively [58]. Le Faouder et al. optimized the sample preparation and the extraction process with extraction using SPE on a C18 cartridge (200 mg, 15 mL; Macherey Nagel), and obtained the separation of 26 oxylipins in human Caco-2 epithelial cells and human primary foam cells [129].

#### 2.3.4. LLE or SPE?

LLE is a widely used method of sample preparation for the extraction of target compounds from aqueous samples; however, when compared with SPE, in samples prepared by LLE a wider peak of phospholipids appeared during the LC–MS analysis [89]. Considering that the SPE method is more suitable for processing a large number of samples, SPE has become a popular method of sample preparation in terms of reproducibility, less use of organic solvents and ease of use. In addition, SPE is very compatible with automatic analysis systems [89]. Although LLE extraction efficiency is usually higher than SPE, many endogenous impurities are extracted using LLE, which can affect separation and quantification [72]. Among the disadvantages of LLE with organic solvents is the poor extraction of hydrophilic compounds, such as PGs and LTs, compared to SPE [53]. SPE has been shown to provide better purification and enrichment than LLE [97]. The disadvantages of SPE are the high cost of SPE cartridges, the fact that it is time-consuming (depending on the procedure used), and the fact that for unstable eicosanoids a long extraction process can be detrimental for further accurate analysis [126].

Often LLE is not used separately, but precedes subsequent SPE to improve the purification of oxylipins [133,134]. Tajima et al. extracted total lipids from the tissues of the right hemisphere of the mouse brain using the Bligh and Dyer method with minor changes and for the further isolation of oxylipins, samples of the aqueous layer were subjected to SPE [135]. However, when comparing the SPE and LLE methods, different authors obtain ambiguous results about how the efficiency of extraction is influenced not only by the choice of solvent for LLE and the cartridge model for SPE. Furthermore, the type of tissue and examined analytes may influence the SPE and LLE efficiency. Sterz et al. compared the LLE and SPE methods for the isolation of eicosanoids from urine and found that LLE allows all analytes to be extracted; however, the extraction efficiency increased with decreasing urine pH (optimal at pH 4). Despite the fact that the signal intensity for some analytes was lower when using LLE than SPE, the best signal-to-noise (S/N) ratios were achieved after LLE over the complete range of analytes [117]. Rago and Fu compared LLE by HAc/IPA/hexane (2:20:30, *v*/*v*/*v*) with SPE (Oasis^®^HLB, Milford, MA, USA) for the extraction of eicosanoids from human and monkey plasma samples. The SPE method showed better recovery and reproducibility than the LLE method [136]. In the case of polar eicosanoids (LTB4, PGD2, PGE2, PGF2α, 13, 14-PGE2 and 8-iso PGF2α), the extraction recovery rate was, on average, 63% enhanced when using SPE [136]. Additionally, when comparing LLE extraction with sodium acetate [99] using six different SPE methods, Ostermann et al. considered LLE as an inappropriate sample preparation for the analysis of oxylipins in plasma [112]. However, Golovko et al. demonstrated that the extraction of PGs from tissue with acetone, followed by LLE, significantly increased the sensitivity level of the LC–MS/MS analysis compared to other extraction methods, due to a significant reduction in the background chemical noise. Dissolving the residue from the lipid extracts of n-hexane:IPA with acetone is less time-consuming and expensive compared to cleaning C18 cartridges. Besides this, PGs are more stable in n-hexane:IPA extracts [49]. Based on the above, in recent years, SPE has more often been chosen for oxylipin analysis than LLE; however, the extraction method finally applied should depend on the studied oxylipin groups. 

Most SPE cartridges and extraction procedures used for the purification of oxylipins are presented in Table 2

#### 2.3.5. New Approaches in Oxylipin Extraction

Although SPE is currently the most widely used method for extracting oxylipins, much attention is paid to the development of solvent-free and miniaturized extraction systems. These new methods include stir-bar-sorptive extraction (SBSE) and liquid-phase microextraction (LPME), but the most popular in oxylipin research is solid-phase microextraction (SPME), used for matrices like blood [93], urine [152] and plasma [153]. The advantages of miniaturization include minimal use of solvents and a small sample volume; however, a very small sample volume can cause problems such as insufficient sensitivity. Typically, SPME and SBSE are used in combination with GC analysis, but they can also be used in combination with LC. LPME can be used with both GC and LC [154,155]. 

Another new solution in the extraction methods of oxylipins is the semi-automatic microextraction by packed sorbents (MEPS) technique. Unlike conventional SPE, the MEPS sorbent bed is integrated into a liquid handling syringe, which allows work with low sample extraction and washing solvent volumes, and manipulations are performed either manually or in combination with laboratory robotics MEPS [156]. Perestrelo et al. showed good selectivity and sensitivity (LOD 0.37 ng/mL and LOQ 1.22 ng/mL) for the measurement of urinary LTB4 using the MEPS technique with a new digitally controlled syringe (eVols) combined with UHPLC [156]. In this study, they compared the performance of the eight MEPS sorbent materials, and porous graphitic carbon (PGC) sorbent was chosen for the MEPS procedure because it provided better extraction efficiency and reproducibility. The study showed that the developed method is accurate and offers simplicity, reduced sample preparation and analysis time, low cost and minimal consumption of extraction solvent compared with traditional methodologies [156]. 

A promising alternative to classical SPE and LLE can be deproteinization using ferromagnetic particles, as this is a fast procedure suitable for a large number of samples. In addition, there is no need for centrifugation, a vacuum or pressure. Suhr et al. demonstrated high-efficiency ferromagnetic particle enhanced deproteination in combination with online SPE for the sample clean-up of seven eicosanoids in human plasma samples. Before protein denaturation using ACN, the ferromagnetic bead suspension was added to the sample. High-speed vortexing leads to the binding of the denatured proteins to the surface of the particles, simultaneously forming a pellet after being placed on the magnetic separator [157]. 

Dried blood spot analysis (DBS) is becoming a popular method. A small drop of whole blood is placed on filter paper and air dried. Then, analytes are removed from the spot by solvent. Benefits include the use of smaller sample volumes (20–25 µL of whole blood), ease of collection, and simplified transport and storage requirements; among the disadvantages are limited stability of the analyzed substances on the filter paper and background interferences from the paper strongly interact with the metabolites [82]. The most common solvent is MeOH. However, the partial pre-addition of water can increase the efficiency of organic extraction by reducing the interaction between the cellulose and the hydroxyl groups of the target analyte with water [158]. Hewawasam et al., during the extraction of DBS with 80% aqueous MeOH, quantified 21 biologically significant oxylipins. This method has a higher LOD compared to other recently described methods, but the LOD value is suitable for a quick and routine analysis of a number of oxylipins in DBS samples [65].

To minimize human intervention and provide high precision, accuracy and throughput in the quantitative determination of eicosanoids, Ferreiro-Vera et al. used an online SPE–LC–MS/MS analysis method based on direct injection of the biofluid into an automated SPE workstation, where sample desalting and deproteinization occur. After this, the resulting eluate is directly injected into the LC–MS analyzer without affecting the electrospray performance [159]. Wagner used the online SPE–LC–MS system equipped with two quaternary pumps and an external Rheodyne MX Series II switching valve, and the UHPLC System and a Strata-X SPE-cartridge (20 × 2.0 mm, 25 µm; Phenomenex, Germany) for the MS detection of 8-iso-PGF2α [160]. Kita et al. used a column-switching reversed-phase LC–MS/MS technique for the quantitation of eicosanoids in murine macrophage-like RAW264.7 cells [161]. Using LC10AD pumps (Shimadzu, Kyoto, Japan), a 3033 autosampler (Shiseido, Tokyo, Japan), and an electrically controlled six-port switching valve, they combined isocratic conditions to achieve three-step gradient separation. They used a tee connector for the online dilution of samples to obtain optimal concentrations of MeOH and FAc, which allowed for the analysis of 14 lipid mediators within 10 min (throughput of 96 samples/24 h) with maximum sensitivity and minimal carryover [161]. Using online SPE–LC–MS/MS analysis, Kortz et al. profiled seven PUFAs and 94 oxidized metabolites in human plasma with a total analysis time of 13 min per sample [91].

Since anion-exchange SPE is not suitable for the extraction of polar lipids such as PG and TX, Sanaki et al. developed a new approach for the analysis of oxidized fatty acids in mouse lung homogenate samples using LC–MS/MS combined with mixed-mode extraction with a spin column [139]. The method is based on the adsorption of oxylipins in neutral conditions on a column (which contained trimethylaminopropyl and octadecyl groups bonded to silica), and all processing procedures (sample loading, washing and elution) were carried out by centrifugation. Mixed-mode SPE allows extraction efficiencies to be obtained of ≥70% for 61 oxylipins and is much more effective compared to RP–SPE. Moreover, the extraction time was reduced from 1 h per six samples to 10 min, and the use of organic solvents was reduced from >20 mL per sample to <1 mL per sample [139]. 

### 2.4. Derivatization Process

The derivatization step is determined by the chosen analytical method. For liquid chromatography (LC) using spectrophotometric and fluorimetric detectors, derivatization allows compounds to be obtained which are sensitive to these types of detectors [162]. Derivatization can also be used as a diagnostic tool to determine which functional groups exist in the oxylipin molecule (hydroxyl groups, carbonyl groups, etc.). 

HPLC coupled with fluorescent detectors (HPLC–FLD) requires analytes to be derivatized into a complex that fluoresces, as these compounds contain no aromatic or naturally fluorescing systems. A simple derivatization reaction with ADAM (9-anthryl diazomethane) and the fluorescent detection of the resultant product (ADAM can be used to derivatize –COOH groups) was originally used for the analysis of PGs and was later used for the derivatization of eicosanoids before HPLC separation [163]. Nithipatikom et al. used SPE followed by derivatization with 2-(2,3-naphthalimino)ethyl trifluoromethanesulfonate (NE-OTf) to determine 14,15-EET, 11,12-EET, and a mixture of 8,9-EET and 5,6-EET from bovine coronary artery endothelial cells using HPLC–FLD [164]. Yue et al., using derivatization with NE-OTf, developed a method for the HPLC–FLD detection of bioactive eicosanoids including PG, DiHETrE, HETE, EET, and ARA from rat cortical brain tissue [126]. 

In the case of HPLC equipped with an ultraviolet detector (HPLC–UV), the analytes must have an active chromophore. Some eicosanoids have specific chromophores such as LTs (which contain conjugated triene) but many eicosanoids do not have any active chromophores (i.e., prostanoids) [97]. Chavis et al. used RP–HPLC with a UV detector (237nm) for the quantification of 5-HETE and 12-HETE in human plasma without derivatization [165]. Aghazadeh-Habashi et al. used derivatization with NE-OTf for the simultaneous quantification of eicosanoids in human plasma and rat heart and kidney by the HPLC method using fluorescence detection [166]. Yue et al. coupled the derivatization of eicosanoids with fluorescence detection using RP–HPLC to determine bioactive PGs, EETs, DiHETEs and HETEs in rat brain tissue, and achieved limits of detection (LODs) and LOQs ranging from 2–20 to 20–70 pg on the column, respectively [126].

Derivatization could lead to a better separation efficacy and improved MS detection. Bollinger et al. describe a new derivatization reagent N-(4-aminomethylphenyl)pyridinium (AMPP). The conversion of the carboxylic acid of eicosanoids to a cationic AMPP amide enabled detection in positive-ESI mode, and manifold improvements in the sensitivity of LC–ESI–MS/MS detection [146]. Meckelmann et al. evaluated whether pentafluorobenzyl (PFB) derivatization and electron capture atmospheric pressure chemical ionization (ECAPCI) (−) or AMPP derivatization and ESI (+) could improve the RP–LC–MS/MS analysis of oxylipins in human plasma, and found that PFB derivatization led to a low sensitivity (LOD~10 nM, 100 fmol on the column, 32 pg on the column) and thus was not suitable for detection by the MS instrumentation used. Compared to AMPP/ESI (+), direct ESI (−) yielded a higher sensitivity for several OH-PUFAs, but it did not result in better analytical performance [167]. This discrepancy with the results obtained by Bollinger et al. may be explained by the different MS instrumentation (Waters triple-quadrupole (QqQ) vs. Sciex QqQ) because the Waters QqQ instruments used (Premier and Quattro) are significantly more sensitive in the positive mode than in the negative ESI mode [167].

GC derivatization is associated with obtaining more volatile compounds, reducing the polarity of functional groups, and as a consequence, improving the chromatographic properties of a substance, or obtaining specific products for a particular type of detector [168]. Some examples of derivatization methods are: N-acylation, methoxyamine formation, esterification, and trimethylsilyl (TMS) ether formation [127]. After the derivatization step, many analytes can be detected simultaneously. However, on the other hand, eicosanoid volatility is increased by derivatization, together with polarity and thermal lability; therefore, GC–MS is applicable mainly to eicosanoids, including the catalytic reduction of highly polar, nonvolatile and thermally labile cysteinyl LTs [18]. In contrast to the quantitative analysis of fatty acids, where only one step of derivatization is required (e.g., methyl, trimethylsilyl or PFB esters of fatty acids), one-step derivatization is not suitable for all oxylipins due to the presence of different functional groups [80]. In the GC–MS and GC–MS/MS analysis of eicosanoids, derivatization with fluorine-rich reagents such as PFB bromide is required, because eicosanoids have two or three thermally labile chemical functionalities, i.e., carboxylic, hydroxylic and keto groups [80,137]. ECAPCI–MS analysis of fatty acyls which have been derivatized as PFB esters is more sensitive compared to ESI analysis of underivatized fatty acyls enabling the efficient normal-phase chiral separation of oxylipins [169]. Knott et al. used esterification with PFB bromide, methoxylation and finally, trimethylsilylation with N, O-bis(trimethylsilyl)-trifluoroacetamide (BSTFA) for GC analysis of PGs in human synovial cells and chondrocyte cultures [170]. Additional derivatization of hydroxyl-groups with N-methyl-trimethylsilyl-trifluoroacetamide (MSTFA) to TMS-ethers made the highly polar oxylipins suitable for GC separation and, at the same time, provided further information for structure elucidation [171]. Fulton found that the conversion of 5,6-δ-lactone to 5,6-DHT permitted convenient derivatization with PFB and more sensitive GC–MS without the need for further purification of biological samples obtained from perfused kidney [60]. However, in the case of LTB4, HPLC–UV and LC–MS, in contrast to GC–MS analysis, a previous derivatization procedure to determine LTB4 in biological fluids is not required, thus increasing recovery and reducing the time for sample pretreatment [156]. The main disadvantages of derivatization are the labor intensity, increase in the total procedure time, risk of thermal decomposition, instability of the derivatives and the lack of reproducibility of the yield of the derivatives, and also loss of analyte due to incomplete interaction, contamination with reagents and undesirable side reactions [78,158,172,173].

## 3. Methods of Oxylipin Analysis

**Figure 4:**
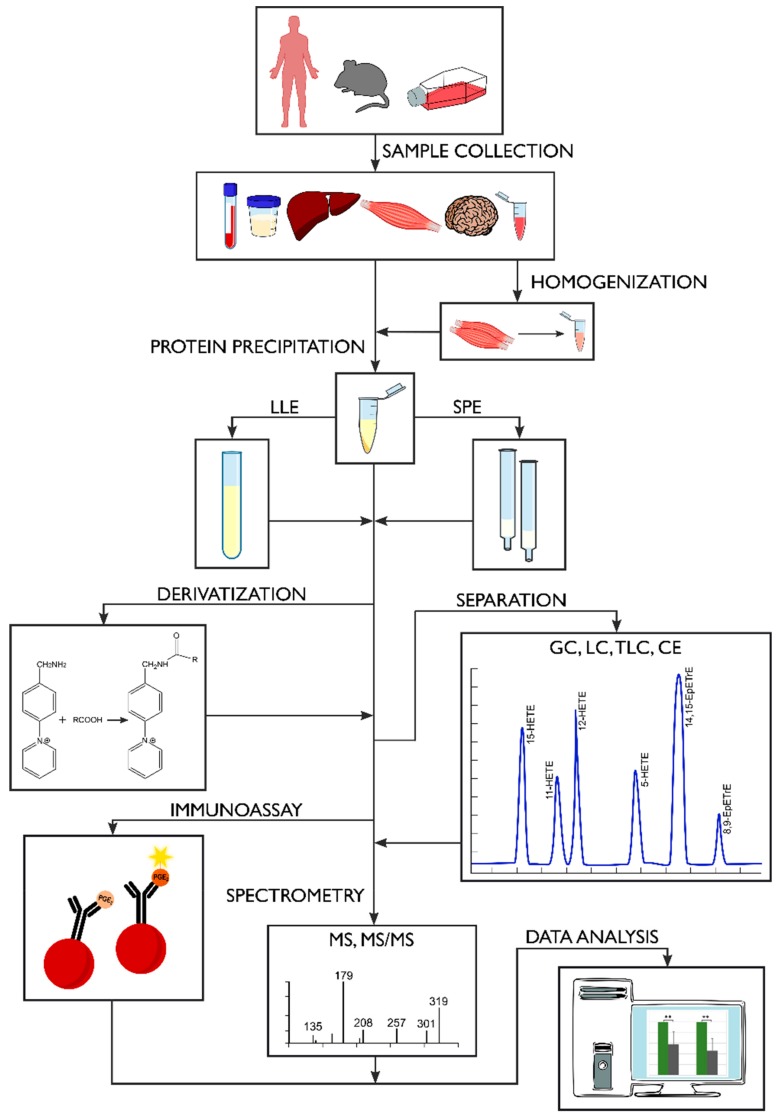
Scheme of the complete analytical procedure of oxylipins.

Presently, there are many methods for measuring the levels of oxylipins in human biological samples. However, they all have certain limitations. This is primarily due to the fact that oxylipins are present in extremely low concentrations in biological matrices, have limited stability and are subject to degradation and auto-oxidation [65]. In addition, many oxylipins, especially those derived from the same original fatty acid, have very similar structures [80]. Therefore, their analysis requires rapid, highly-sensitive and accurate analytical methods [112,174], see Figure 4. 

Various methods, including immunoassay, thin-layer chromatography (TLC), HPLC–UV, HPLC–FLD, GC–MS and LC–MS were used to analyze oxylipins. However, some of these methods are not specific or sensitive enough, and often require derivatization [53]. Currently, more often GC–MS and LC–MS are used for oxylipin determination. GC–MS has long been a common analytical technique for the quantitative and structural interpretation of eicosanoids; however, due to the cost of equipment and the difficulty in preparing samples, fewer laboratories use GC–MS [127]. GC–MS requires purification steps after derivatization to remove impurities formed during the derivatization process, which can make the analysis laborious, expensive and time-consuming [59]. Among the less frequently used techniques for the analysis of oxylipins, capillary electrophoresis with a photodiode array detector (CE–UV) can be noted, which was sufficiently sensitive to detect and measure EET and DHET enantiomers from murine liver (unlike chiral-phase HPLC) [111,175]. The most popular method for detecting oxylipins is LC–MS. One of the reasons is that MS allows quantitative determination at very low levels in complex matrices. LC-MS gives a better separation of the isomers compared with HPLC–UV and immunoassay [97].

## 4. Conclusions

Oxylipins play an important role in various biologic processes. A large number of enzymatic and non-enzymatic pathways lead to the formation of hundreds of oxylipins. Effective simultaneous determination of a large number of compounds is possible only during sample preparation by the PP, LLE and SPE methods, both individually and jointly. Modern sample preparation techniques make the analysis more economical and less invasive and time-consuming.

## Figures and Tables

**Table 1:** Selected oxylipins and their physiopathological functions in human studies.

Disease	Oxylipin	Precursor	Direction of Change	Function	Ref.
Obesity	5-, 11-, 20-HETE	ARA	↑	promotion of inflammation, blood pressure regulation	[10,20,21]
	15-HETE	ARA	↑	substrates for lipoxins synthesis	
	12,13-DiHOME	LA	↓	brown adipose tissue lipid uptake activation	
	12,13-Di/EpOME	LA	↓	putative markers of adipose lipolysis	
	15-HETrE	DGLA	↑	antiproliferative function	
	5-, 8-, 12-HETE	ARA	↑	associated with low-grade inflammation	[22]
	PGD2	ARA	↑	polarization of adipose tissue macrophage against inflammation	[23]
Metabolic syndrome	20-HETE	ARA	↑	vascular inflammation, angiogenesis	[15,24]
	F2-isoprostanes	ARA	↑	oxidative stress marker	
	LXA4	ARA	↓	promotion of inflammation resolution	[25]
Type II diabetes	8-iso-PGF2α	ARA	↑	oxidative stress marker	[26,27]
	11,12-, 14,15-DiHETrE	ARA	↑	EpETrE’s less active metabolites	[28,29,30]
	13-oxo-ODE	LA	↑	inhibition of inflammation	
	11(12)-, 14(15)-EpETrE	ARA	↑	vasodilation	
	9(10)-EpOME	LA	↑	leukotoxin	
	12(13)-EpOME	LA	↑	putative marker of adipose lipolysis	
	9(10)-EpODE	ALA	↑	putative markers of adipose lipolysis	
Hypothyroidism	PGI2	ARA	↑	platelet activation inhibitor	[31]
	PGE2	ARA	↓	promotion of arterial thrombosis	
	12-HETE	ARA	↓	blood pressure regulation	
Hyperthyroidism	12-HETE	ARA	↑	blood pressure regulation	[31]
	20-HETE	ARA	↑	vasoconstriction	
Sepsis	11-HETE	ARA	↓	promotion of inflammation	[32]
	PGE2	ARA	↓	vasodilation	
	TXB2	ARA	↓	downstream metabolite of TXA2 which is involved in platelet aggregation and vasoconstriction	
Achilles tendopathy	13-HODE	LA	↑	association with pain	[33]
	12,13-DiHOME	LA	↑	association with pain	
Coronary artery disease	9-HETE	ARA	↑	oxidative stress marker	[16]
	F2-isoprostanes	ARA	↑	oxidative stress marker	
Myocardial infraction	11-dehydro-TXB2	ARA	↑	oxidative stress marker	[17]
	2,3-dinor-TXB2	ARA	↑	oxidative stress marker	
Atherosclerosis	9-HODE in LDL	LA	↑	lipid peroxidation marker	[34]
Acute respiratory distress syndrome	9(10)-EpOME	LA	↑	leukotoxin	[35]
Asthma	PGE2	ARA	↑	promotion of inflammation	[11,36]
	PGI2	ARA	↑	inhibition of thrombosis and inflammation	
	TXB2	ARA	↑	promotion of inflammation	
	PGF2α	ARA	↓	promotion of inflammation	
	6-keto-PGF1α	ARA	↓	oxidative stress marker	
Cystic fibrosis	LXA4	ARA	↓	inhibition of inflammation	[13]
	RvE1	EPA	↓	promotion of inflammation resolution, positively associated with better lung function	
Alzheimer’s disease	total HODE	LA	↑	in vivo lipid peroxidation marker	[37]
Schizophrenia	8-iso-PGF2α	ARA	↑	oxidative stress marker	[38]
Breast cancer	9-, 13-HODE	LA	↑	PPAR-γ ligand	[14,39,40,41]
	9-, 13-HOTrE	LA	↑	inhibition of inflammation	
	12-HHTrE	ARA	↑	polymorphonuclear leucocytes (PMN) chemotaxis enhancer	
Colorectal cancer	2,3-dinor-PGF2α	ARA	↑	oxidative stress marker	[42]
	19-HETE	ARA	↑	possible competitive antagonist of 20-HETE	
	12-keto-LTB4	ARA	↑	inactive metabolite of pro-inflammatory LTB4	
	9-HODE	LA	↓	PPAR-γ ligand	
	13-HODE	LA	↓	promotion of apoptosis	
	PGE2	ARA	↑	promotion of inflammation	[12,36]
	PGI2	ARA	↓	inhibition of thrombosis and inflammation	
Non-small cell lung cancer	15S-HETE	ARA	↓	induction of apoptosis	[43]
	13S-HODE	LA	↓	induction of apoptosis	
Prostate cancer	LTB4	ARA	↑	promotion of survival and proliferation of cancerous cells	[44]

LDL, Low density lipoprotein; PMN, Polymorphonuclear neutrophil; PPAR-γ, Peroxisome proliferator-activated receptor γ.

**Table 2:** Representative SPE cartridges and extraction conditions for purification of selected oxylipins.

Sample	Stationary Phase	Sample Preparation	Column Precondition	Sample Wash	Elution	Reference
OCTADECYL PHASES						
Human plasma (1 mL) Rat Carrageenan-induced air pouch fluid (1 mL)	Bond Elut C18	Pouch fluid: + 1 mL heparinized saline centrifugation at 1000× *g*, 4 °C, 10 min	2 mL MeOH 2 mL H_2_O	2 mL MeOH 2 mL petroleum ether	1 mL methyl formate	[137]
Rat brain (~500 mg) rat liver (~500 mg) plasma (500 µL)	C18-E (6 mL, 500 mg)	Tissues: homogenization in H_2_O, adjusted to 15% MeOH to 3 mL incubation on ice, 30 min centrifugation at 3000 rpm, 5 min + 0.025 mM HCL (pH 3) Plasma: dilution with H_2_O, adjusted to 15% MeOH to 3 mL incubation on ice, 30 min centrifugation at 3000 rpm, 5 min + 0.025 mM HCl (pH 3)	20 mL MeOH 20 mL H_2_O	20 mL 15% MeOH 20 mL H_2_O 10 mL Hex	15 mL methyl formate	[113]
Rat serum (400 µL) rat mammary tumor (~200 mg)	Bakerbond C18 (3 mL, 500 mg)	Serum: + 0.5 mL MeOH + 4 mL MeOH Tumor: homogenization with 2 mL H_2_O on ice 4 °C, 30 min centrifugation at 3000 rpm, 5 min	10 mL MeOH 10 mL H_2_O	2 mL H_2_O 2 mL 10% MeOH	3 × 0.5 mL MeOH	[131]
Human phagocytes culture supernatant	Waters C18	1:10 dilution with H_2_O (pH ~3.5)	1× MeOH 2× H_2_O	1× H_2_O 1× Hex	6 mL methyl formate	[138]
Human plasma (300 µL)	Sep-Pak C18 (2.8 mL, 500 mg)	+ 1.5 mL MeOH/ACN (1/1, *v*/*v*) 4 °C, overnight centrifugation at 400× *g*, 20 mindilution with H_2_O to 10% MeOH/ACN	2 mL MeOH 2 mL 0.1% HAc	sample + 2 mL 0.1% HAc	2 mL 0.1% HAc in MeOH	[114]
			10 mL ethanol 20 mL H_2_O	sample acidified with 0.1 M HCl 10 mL H_2_O 1 mL 35% ethanol	2 mL ethanol	
			10 mL methanol 10 mL H_2_O	sample acidified with 0.1 M HCl 5 mL 15% MeOH 5 mL H_2_O 2.5 mL Hex	2 mL Hex	
Mouse brain tissue (20 mg)	Sep-Pak C18	microwave processing homogenization in 3 mL 15% MeOH, 0.005% BHT (pH 3) centrifugation at 2000× *g*, 4 °C, 10 min		20 mL 15% MeOH 20 mL H_2_O dried with syringe air	10 mL methyl formate	[49]
Mouse lung homogenate (50 µL)	Sep-Pak tC18 (6 mL, 500 mg)	+ 0.45 mL MeOH, 10 min vortex 0.5 mL supernatant collected + 4.5 mL HCL in H_2_O (pH 3.5)	12 mL MeOH 12 mL H_2_O	12 mL H_2_O 6 mL Hex	9 mL methyl formate	[139]
	MonoSpin™ C18	+ 0.45 mL MeOH, 10 min vortex centrifugation at 9000× *g*, 4 °C, 5min evaporation with N_2_, 40 °C reconstituted in 0.1 mL MeOH + 0.9 mL HCl in H_2_O (pH 3.5)	0.3 mL MeoH 0.3 mL H_2_O	centrifugation at 9000× *g*, 4 °C, 1 min 0.3 mL H_2_O 0.3 mL Hex centrifugation at 9000× *g*, 4 °C, 1 min	2 × 0.3 mL methyl formate	
Rat hypothalamus (95 mg)	Hypersep C18 (3 mL, 500 mg)	homogenization in 2 mL water acidified with 1 M HCL to pH 3.0 centrifugation at 7000 rpm	6 mL MeOH 6 mL H_2_O	2 mL 2% HAc	2 mL MeOH	[140]
OCTYL PHASES						
Human plasma (1 mL)	Discovery DSC-C8		1 mL MeOH 1 mL 0.1% FAc	1 mL 0.1% FAc	1 mL MeOH	[141]
POLYMERIC PHASES						
Human plasma (500 µL)	Bond Elut Certify II (3 mL, 200 mg)	+ 10 µL 0.2 mg/mL BHT, EDTA, 100 µM indomethacin, 100 µM soluble epoxide hydrolase inhibitor trans-4-[4-(3-adamantan-1-yl-ureido)-cyclohexyloxy]-benzoic acid in 50% MeOH + 1.4 mL ice cold MeOH, −80 °C, 30 min 10 min, 4 °C, 20,000× *g* N2 to final volume <1 mL + 2 mL 0.1 M disodium hydrogen phosphate buffer (pH 5.5)	1 × 1% HAc in EA/n-Hex (75/25, *v*/*v*) 1× MeOH 1 × 0.1 M disodium hydrogen phosphate buffer (pH 6)	3 mL H_2_O 3 mL 50% MeOH dried under N_2_, 1 min	2 mL 1% HAc in EA/n-Hex (75/25, *v*/*v*)	[59]
Rat brain/heart/kidney/liver/lung/pancreas/red blood cells (50 mg) plasma (200 μL)	Bond Elut Certify II	+ 0.5 mL distilled H_2_O + 0.5 mL MeOH + 300 μL (100 μL for plasma) 10 M NaOH, 60 °C, 20 min + 300 μL 60% HAc + 2 mL 1 M sodium acetate buffer (pH 6) adjusted to pH 6 centrifugation	2 mL MeOH 2 mL 5% MeOH, 0.1 M sodium acetate (pH 7)	2 mL MeOH/H_2_O (1/1, *v*/*v*)	2 mL Hex/EA (75/25, *v*/*v*)	[9]
Human whole-blood and platelet-rich plasma (250 μL)	HyperSep Retain PEP	dilution with 5% MeOH, 0.1% HAc with 0.009 mM BHT	5% MeOH, 0.1% HAc	2 × 5% MeOH, 0.1% HAc	1 mL EA 1 mL MeOH	[122]
Human milk (1.5–2 mL)	Oasis HLB (60 mg, 30 µm)	centrifugation + 10 µL 0.2 mg/mL BHT/EDTA	4 mL EA 8 mL MeOH 8 mL 5% MeOH, 0.1% HAc	8 mL 5% MeOH, 0.1% HAc	2 mL MeOH 3 mL ACN 2 mL EA	[124]
Human plasma (250 µL)	Oasis HLB (60 mg, 30 µm)	+ 0.2 mg BHT/EDTA	0.5 mL MeOH		2 mL EA	[51]
Human plasma (50–200 μL)	Oasis HLB (3 mL, 60 mg)	Free oxylipins: 200 µL plasma + 10 µL 0.2 mg/mL of BHT and TPP and 1 mg/mL EDTA in 50% MeOH + 1 mL 5% MeOH, 0.1% FAc Total oxylipins: 50–100 µL plasma + 10 µL 0.2 mg/mL of BHT and TPP and 1 mg/mL EDTA in 50% MeOH + 0.1 mL 0.1% acetic acid and 0.1% BHT in MeOH 80 °C, overnight + 0.2 mL of 0.25 M sodium carbonate solution, 60 °C, 30 min, constant shaking + 25 µL HAc, 1.575 mL H_2_O to pH 4–6	1× EA 1× MeOH 2 × 5% MeOH, 0.1% FAc	2 × 5% MeOH, 0.1% FAc dried under ~20 psi, 20 min	0.5 mL MeOH 1.5 mL EA into 6 µL of 30% glycerol in MeOH	[64]
Human plasma (200 µL)	Oasis HLB 96-well plates (30 mg)		1 mL MeOH 1 mL H_2_O	1.5 mL 5% MeOH	1.2 mL MeOH	[142]
Human serum/plasma/washed platelets (200 μL)	Oasis HLB (1 mL, 10 mg)	+ 1 mL MeOH centrifugation at 20,000× *g*, 4 °C, 10 min	0.15 mL MeOH 1 mL 0.03% FAc in H_2_O	1 mL 0.03% FAc in H_2_O 1 mL 15% ethanol, 0.03% FAc in H_2_O 3 mL petroleum ether	0.2 mL MeOH	[143]
Human serum (500 µL) Human sputum (500 µL) Human bronchoalveolar lavage fluid (500 µL)	Oasis HLB 3 mL, 60 mg	+ 1.5 mL 5% HAc	2 mL MeOH 2 mL 0.1% HAc	2 mL 0.1% HAc	2 mL MeOH	[144]
Human serum (950 µL)	Oasis HLB (60 mg)	+ 10 mL 0.2 mg/mL BHT and EDTA	1× EA 1× MeOH	2 × 5% MeOH, 0.1% HAc dried under 0.2 bar	500 μL MeOH 1500 μL EA	[76]
Human urine	Oasis HLB (3 mL, 60 mg)	dilution with H_2_O to 2 mL + 0.5 mL 0.5% HAc	3 mL H_2_O 3 mL 0.1% HAc	3 mL 0.1% HAc dried at −30 kPa, 30 min	3 mL ACN	[116]
		dilution with H_2_O to 2.7 mL + 0.3 mL 1% HAc	3 mL H_2_O 3 mL 0.1% HAc	1 mL 50% MeOH 3 mL 0.1% HAc dried at −30 kPa, 30 min	1 mL MeOH	
Human cerebrospinal fluid (0.11–1 mL) Rat cortical brain tissue	Oasis HLB (30 mg)	cerebrospinal fluid: dilution with 0.12 M potassium phosphate buffer with 5 mM magnesium chloride, 0.113 mM BHT Tissue: + 0.12 M potassium phosphate buffer with 5 mM magnesium chloride, 0.113 mM BHT centrifugation at 10,000 rpm, 30 min	1 mL MeOH 1 mL H_2_O	3 × 1 mL 5% MeOH	MeOH	[145]
Mouse serum (1–10 µL) Human lung epithelial cells (5 × 104 cells/well) Rat fibroblast cell line culture medium (50 µL)	Oasis HLB (10 mg)	+ 2× MeOH vortex, 10 s dilution with H_2_O to 10% MeOH	1 mL MeoH 2 × 0.75 mL 5% MeOH	2 × 1 mL 5% MeOH	1 mL MeOH	[146]
Cow heart (~130 mg) cow liver (~320 mg) Pig/elk/cow brain (~80–180 mg) Human plasma (250 µL) Human milk (500 µL) Cell medium 2 mL	Oasis HLB (60 mg, 30 µm)	Tissues: homogenization in 1 mL MeOH with 10 µL 0.2 mg/mL BHT/EDTA in 50% MeOH centrifugation at 2125× *g*, 10 min diluted to 5% MeOH Plasma, milk, cell medium: + 10 µL 0.2 mg/mL BHT/EDTA in 50% MeOH	2 mL EA 2 × 2 mL MeOH 2 mL 5% MeOH, 0.1% HAc	2 × 2 mL 5% MeOH, 0.1% HAc	3 mL ACN 2 mL MeOH 1 mL EA	[75]
Mouse serum/bronchoalveolar lavage fluid (250 µL)	Oasis HLB (60 mg)	+ 0.2% *w*/*w* TPP/BHT	2 mL EA 2 × 2 mL MeOH 2 mL 5% MeOH, 0.1% HAc	1.5 mL 5% MeOH, 0.1% HAc dried under vacuum, 20 min	0.5 mL MeOH 2 mL EA	[47]
Rat cortical brain tissue (20 mg)	Oasis HLB (30 mg)	homogenization with 0.2 mL MeOH, 0.4 µL FAc on ice centrifugation at 14,000 rpm, 0 °C, 10 min + 1.8 mL H_2_O	1 mL MeOH 1 mL acetone 2 mL Hex 1 mL acetone 1 mL MeOH 2 mL H_2_O	3 mL H_2_O 1 mL 10% MeOH dried under Ar pressure, 10 min	2 mL ACN	[126]
Rat kidney (100 mg)	Oasis	homogenization in 0.2 mL MeOH with 0.01 M BHT and 5 µL FAc centrifugation at 14,000 rpm, 0 °C, 15 min dilution with H_2_O to 2 mL	2 mL 0.1% FAc 2 mL MeOH 2 mL EA	2 mL 0.1% FAc 2 mL 10% MeOH, 0.1% FAc	1.5 mL 0.1% FAc with 0.01 BHT 0.5 mL MeOH, 0.2% FAc with 0.01 BHT	[128]
Human plasma/urine (1 mL)	Oasis MAX	Urine: + 1 mL 40 mM FAc (pH 2.6) Plasma: + 1 mL 1M KOH in MeOH, 37 °C, 30 min + 1 mL MeOH + 0.2 mL 5 M HCL + 1.7 mL 40 mM FAc to pH 2.6 centrifugation at 10,000× *g*, 4 °C, 10 min	2 mL MeOH 2 mL 20 mM FAc	2 mL 2% ammonium hydroxide 2 mL MeOH/20 mM FAc (40/60, *v*/*v*) 2 mL Hex 2 mL Hex/EA (70/30, *v*/*v*)	EA	[147]
Human plasma (200 µL)	Strata-X (3 mL, 60 mg, 33 µm)	+ 0.8 mL H_2_O, pH 3 acidified with HCL to pH 3	2 mL MeOH 2 mL H_2_O, pH 3	10% MeOH in H_2_O pH 3	2 mL MeOH	[148]
Human plasma (500 µL)	Strata-X (3 mL, 200 mg, 33 µm)	+ 1.5 mL 90% MeOH centrifugation at 6000 rpm, 10 min	3 mL MeOH 3 mL H_2_O	3 mL H_2_O	3 mL MeOH	[19]
Human plasma (200 µL)	Strata-X (6 mL, 200 mg, 33 µm)	+ 0.5 mL cold MeOH with 20 mg/mL BHT/EDTA −80 °C, 30 min centrifugation at 14,000 rpm, 4 °C, 10 min	6 mL MeOH 6 mL H_2_O	6 mL 10% MeOH air-dried, 2 min	6 mL MeOH with 0.0004% *w*/*v* BHT	[53]
Human whole blood	Strata-X 96-well plates (60 mg/well, 33 μm)	1:1 dilution with RPMI-1640 medium (+ 25 mM Hepes and l-glutamine) + calcium ionophore A23187 (final concentration 30 μM), 37 °C, 30 min centrifugation at 1300 rpm, 10 min + 10% MeOH to 1 mL	MeOH H_2_O	10% MeOH	1.0 mL MeOH	[149]
Control human plasma (20 µL) Mouse and human tissue: adipose, liver, muscle (2 mg)	Strata-X (3 mL, 60 mg)	Plasma: + 1 mL phosphate salt buffer Tissues: homogenization in 10% MeOH	3 mL MeOH 3 mL H_2_O	10% MeOH	1 mL MeOH	[72]
Cell culture, cell medium (2 mL)	Strata-X	Medium: + 0.1 mL ethanol centrifugation at 3000 rpm, 5 min Cells: + 0.5 mL MeOH + 1 mL phosphate-buffered saline centrifugation at 3000 rpm, 5 min	2 mL MeOH 2 mL H_2_O	1 mL 10% MeOH	1 mL MeOH	[132]
ANION-EXCHANGE PHASES						
Zebrafish embryo	Strata-X-A (3 mL, 200 mg, 22 µm)	saponification	3 mL MeOH 3 mL H_2_O	4 mL MeOH—cholesterol 3 × 6 mL air 4 mL ACN—α-tocopherol	4 mL FAc/MeOH/ACN (5/47.5/47.5)	[150]
Human urine	Strata-X-AW (3 mL, 100 mg)	+ 200 mM MeOH/HCl centrifugation at 10,000 rpm, 5 min	2 mL MeOH 2 mL H_2_O	4 mL H_2_O	1 mL MeOH	[151]
VARIOUS PHASES						
Human urine (2 mL)	Bond Elut C18 (3 mL, 500 mg)	+ 11.25 mL MeOH/CH3Cl (2:1, *v*/*v*), room temperature, 1 h + 3.75 mL CH3Cl + 3.75 mL water centrifugation at 2500 rpm, 10 min CH3Cl phase evaporated and reconstituted in 100 μL MeOH	5 mL MeOH 5 mL H_2_O	5 mL H_2_O 3 mL 5% MeOH dried under vacuum	4 mL MeOH	[117]
	Oasis HLB (6 mL, 500 mg)		5 mL MeOH 5 mL ACN 5 mL H_2_O	3 mL 5% ACN dried under vacuum	4 mL ACN	
	Strata-X (6 mL, 200 mg)		5 mL MeOH 5 mL H_2_O	3 mL 10% MeOH	4 mL MeOH	
	Chromabond Easy (6 mL, 200 mg) Oasis MAX (6 mL, 500 mg)		5 mL 2% FAc in MeOH 5 mL H_2_O	5 mL H_2_O 3 mL 25% MeOH 3 mL ACN dried under vacuum	2 × 2 mL MeOH	
Mouse colon tissue Human epithelial colorectal adenocarcinoma cells Cell supernatant	Marchery Nagel C18 (15 mL, 200 mg)	Colon tissue: homogenization in 0.5 mL of HBSS + 1 mL MeOH centrifugation at 900× *g*, 4 °C, 15 min Cells/supernatant: + 1 mL MeOH centrifugation at 900× *g*, 4 °C, 15 min	10 mL MeOH 10 mL 0.02 M HCL/MeOH (90/10, *v*/*v*)	5 mL 0.02 M HCl/MeOH (90/10, *v*/*v*) dried under aspiration	5 mL methyl formate	[129]
Foam macrophages supernatant Mouse peritoneal exudate	Oasis HLB 96-well plates	centrifugation at 20,000× *g*, 4 °C, 20 min	4 mL MeOH 4 mL H_2_O	2 mL 5% MeOH 2 mL 10% MeOH 2 mL H_2_O dried under aspiration, 15 min centrifugation at 200× *g*, 2 min	4 mL methyl formate	
Human plasma (500 μL)	Oasis HLB (3 mL, 60 mg, 30 μm)	1:1 dilution with 5% MeOH acidified with 0.1% HAc centrifugation at 20,000× *g*, 4 °C, 10 min	1× EA 1× MeOH 2 × 5% MeOH, 0.1% HAc	2 × 5% MeOH, 0.1% HAc	0.5 mL MeOH 1.5 mL EA	[112]
		1:1 dilution with 40% MeOH centrifugation at 20,000× *g*, 4 °C, 10 min	1× EA 1× MeOH 1 × 20% MeOH, 0.1% FAc	1 × 20% MeOH, 0.1% FAc	2.0 mL MeOH	
	SepPak tC18 (6 mL, 500 mg, 37–55 µm)	+ 1.5 mL 20% MeOH centrifugation at 20,000× *g*, 4 °C, 10 min + 80 µL conc. HAc to pH 3	3× MeOH 3× H_2_O	10 mL H_2_O 6 mL Hex	8 mL methyl formate	
	Bond Elut Certify II (3 mL, 200 mg, 47–60 µm)	+ 500 μL 1 M sodium acetate buffer (pH 6) centrifugation at 20,000× *g*, 4 °C, 10 min	1× MeOH 1 × 0.1 M sodium acetate buffer, 5% MeOH	1× MeOH/H_2_O (50/50, *v*/*v*)	2.0 mL n-Hex/EA (25/75, *v*/*v*)	
					2.0 mL n-Hex/EA (75/25, *v*/*v*)	
	Strata-X (3 mL, 100 mg, 33 µm)	1:1 dilution with 20% MeOH centrifugation at 20,000× *g*, 4 °C, 10 min	3.5 mL MeOH 3.5 mL H_2_O	3.5 mL 10% MeOH	1.0 mL MeOH	

ACN: Acetonitrile; BHT: Butylated hydroxytoluene; EA: Ethyl acetate; EDTA: Ethylenediaminetetraacetic acid; FAc: Formic acid; HAc: Acetic acid; MeOH: Methanol; TPP: Triphenylphosphine.

## References

[1] Gabbs M., Leng S., Devassy J.G., Monirujjaman M., Aukema H.M. (2015). Advances in Our Understanding of Oxylipins Derived from Dietary PUFAs. Adv. Nutr. An Int. Rev. J..

[2] Vigor C., Bertrand-Michel J., Pinot E., Oger C., Vercauteren J., Le Faouder P., Galano J.M., Lee J.C.Y., Durand T. (2014). Non-enzymatic lipid oxidation products in biological systems: ASSESSMENT of the metabolites from polyunsaturated fatty acids. J. Chromatogr. B Anal. Technol. Biomed. Life Sci..

[3] Shearer G.C., Walker R.E. (2018). An overview of the biologic effects of omega-6 oxylipins in humans. Prostaglandins Leukot. Essent. Fat. Acids.

[4] Khan S.A., Ali A., Khan S.A., Zahran S.A., Damanhouri G., Azhar E., Qadri I. (2014). Unraveling the Complex Relationship Triad between Lipids, Obesity, and Inflammation. Mediators Inflamm..

[5] Yeung J., Hawley M., Holinstat M. (2017). The expansive role of oxylipins on platelet biology. J. Mol. Med..

[6] Christophersen O.A., Haug A. (2011). Animal products, diseases and drugs: a plea for better integration between agricultural sciences, human nutrition and human pharmacology. Lipids Health Dis..

[7] Spector A.A., Kim H.-Y. (2015). Cytochrome P450 epoxygenase pathway of polyunsaturated fatty acid metabolism. Biochim. Biophys. Acta - Mol. Cell Biol. Lipids.

[8] Yang J., Dong H., Hammock B.D. (2011). Profiling the regulatory lipids: Another systemic way to unveil the biological mystery. Curr. Opin. Lipidol..

[9] Arnold C., Markovic M., Blossey K., Wallukat G., Fischer R., Dechend R., Konkel A., Von Schacky C., Luft F.C., Muller D.N. (2010). Arachidonic acid-metabolizing cytochrome P450 enzymes are targets of ω-3 fatty acids. J. Biol. Chem..

[10] Austin C., Sordillo L.M., Zhang C., Fenton J.I. (2017). Obesity is positively associated with arachidonic acid-derived 5- and 11-hydroxyeicosatetraenoic acid (HETE). Metabolism.

[11] Zhou J., Chen L., Liu Z., Sang L., Li Y., Yuan D. (2018). Changes in erythrocyte polyunsaturated fatty acids and plasma eicosanoids level in patients with asthma. Lipids Health Dis..

[12] Rigas B., Goldman I.S., Levine L. (1993). Altered eicosanoid levels in human colon cancer. J. Lab. Clin. Med..

[13] Yang J., Eiserich J.P., Cross C.E., Morrissey B.M., Hammock B.D. (2012). Metabolomic profiling of regulatory lipid mediators in sputum from adult cystic fibrosis patients. Free Radic. Biol. Med..

[14] Kumar N., Gupta G., Anilkumar K., Fatima N., Karnati R., Reddy G.V., Giri P.V., Reddanna P. (2016). 15-Lipoxygenase metabolites of α-linolenic acid, [13-(S)-HPOTrE and 13-(S)-HOTrE], mediate anti-inflammatory effects by inactivating NLRP3 inflammasome. Sci. Rep..

[15] Tsai I.-J., Croft K.D., Mori T.A., Falck J.R., Beilin L.J., Puddey I.B., Barden A.E. (2009). 20-HETE and F2-isoprostanes in the metabolic syndrome: The effect of weight reduction. Free Radic. Biol. Med..

[16] Shishehbor M.H., Zhang R., Medina H., Brennan M.-L., Brennan D.M., Ellis S.G., Topol E.J., Hazen S.L. (2006). Systemic elevations of free radical oxidation products of arachidonic acid are associated with angiographic evidence of coronary artery disease. Free Radic. Biol. Med..

[17] Foegh M.L., Zhao Y., Madren L., Rolnick M., Stair T.O., Huang K.S., Ramwell P.W. (1994). Urinary thromboxane A 2 metabolites in patients presenting in the emergency room with acute chest pain. J. Intern. Med..

[18] Tsikas D. (1998). Application of gas chromatography-mass spectrometry and gas chromatography-tandem mass spectrometry to assess in vivo synthesis of prostaglandins, thromboxane, leukotrienes, isoprostanes and related compounds in humans. J. Chromatogr. B Biomed. Appl..

[19] Berkecz R., Lísa M., Holčapek M. (2017). Analysis of oxylipins in human plasma: Comparison of ultrahigh-performance liquid chromatography and ultrahigh-performance supercritical fluid chromatography coupled to mass spectrometry. J. Chromatogr. A.

[20] Lynes M.D., Leiria L.O., Lundh M., Bartelt A., Shamsi F., Huang T.L., Takahashi H., Hirshman M.F., Schlein C., Lee A. (2017). The cold-induced lipokine 12,13-diHOME promotes fatty acid transport into brown adipose tissue. Nat. Med..

[21] Xi S., Pham H., Ziboh V.A. (2000). 15-Hydroxyeicosatrienoic acid (15-HETrE) suppresses epidermal hyperproliferation via the modulation of nuclear transcription factor (AP-1) and apoptosis. Arch. Dermatol. Res..

[22] Möller K., Ostermann A.I., Rund K., Thoms S., Blume C., Stahl F., Hahn A., Schebb N.H., Schuchardt J.P. (2016). Influence of weight reduction on blood levels of C-reactive protein, tumor necrosis factor-α, interleukin-6, and oxylipins in obese subjects. Prostaglandins Leukot. Essent. Fat. Acids.

[23] Virtue S., Masoodi M., de Weijer B.A.M., van Eijk M., Mok C.Y.L., Eiden M., Dale M., Pirraco A., Serlie M.J., Griffin J.L. (2015). Prostaglandin profiling reveals a role for haematopoietic prostaglandin D synthase in adipose tissue macrophage polarisation in mice and humans. Int. J. Obes..

[24] Hoopes S.L., Garcia V., Edin M.L., Schwartzman M.L., Zeldin D.C. (2015). Vascular actions of 20-HETE. Prostaglandins Other Lipid Mediat..

[25] Yu D., Xu Z., Yin X., Zheng F., Lin X., Pan Q., Li H. (2015). Inverse Relationship between Serum Lipoxin A4 Level and the Risk of Metabolic Syndrome in a Middle-Aged Chinese Population. PLoS ONE.

[26] Liu J.-B., Li W.-J., Fu F.-M., Zhang X.-L., Jiao L., Cao L.-J., Chen L. (2015). Inverse correlation between serum adiponectin and 8-iso-prostaglandin F2α in newly diagnosed type 2 diabetes patients. Int. J. Clin. Exp. Med..

[27] Mukhtar M.H., El-Emshaty H.M., Alamodi H.S., Nasif W.A. (2016). The Activity of Serum 8-Iso-Prostaglandin F2α as Oxidative Stress Marker in Patients with Diabetes Mellitus Type 2 and Associated Dyslipidemic Hyperglycemia. J. Diabetes Mellit..

[28] Grapov D., Adams S.H., Pedersen T.L., Garvey W.T., Newman J.W. (2012). Type 2 Diabetes Associated Changes in the Plasma Non-Esterified Fatty Acids, Oxylipins and Endocannabinoids. PLoS ONE.

[29] Altmann R., Hausmann M., Spöttl T., Gruber M., Bull A.W., Menzel K., Vogl D., Herfarth H., Schölmerich J., Falk W. (2007). 13-Oxo-ODE is an endogenous ligand for PPARγ in human colonic epithelial cells. Biochem. Pharmacol..

[30] Caligiuri S.P.B., Parikh M., Stamenkovic A., Pierce G.N., Aukema H.M. (2017). Dietary modulation of oxylipins in cardiovascular disease and aging. Am. J. Physiol. Circ. Physiol..

[31] Yao X., Sa R., Ye C., Zhang D., Zhang S., Xia H., Wang Y., Jiang J., Yin H., Ying H. (2015). Effects of thyroid hormone status on metabolic pathways of arachidonic acid in mice and humans: A targeted metabolomic approach. Prostaglandins Other Lipid Mediat..

[32] Bruegel M., Ludwig U., Kleinhempel A., Petros S., Kortz L., Ceglarek U., Holdt L.M., Thiery J., Fiedler G.M. (2012). Sepsis-associated changes of the arachidonic acid metabolism and their diagnostic potential in septic patients*. Crit. Care Med..

[33] Gouveia-Figueira S., Nording M.L., Gaida J.E., Forsgren S., Alfredson H., Fowler C.J. (2015). Serum levels of oxylipins in achilles tendinopathy: An exploratory study. PLoS ONE.

[34] Jira W., Spiteller G., Carson W., Schramm A. (1998). Strong increase in hydroxy fatty acids derived from linoleic acid in human low density lipoproteins of atherosclerotic patients. Chem. Phys. Lipids.

[35] Ozawa T., Sugiyama S., Hayakawa M., Satake T., Taki F., Iwata M., Taki K. (1988). Existence of Leukotoxin 9,10-Epoxy-12-Octadecenoate in Lung Lavages from Rats Breathing Pure Oxygen and from Patients with the Adult Respiratory Distress Syndrome. Am. Rev. Respir. Dis..

[36] Wang D., Dubois R.N. (2006). Prostaglandins and cancer. Gut.

[37] Yoshida Y., Yoshikawa A., Kinumi T., Ogawa Y., Saito Y., Ohara K., Yamamoto H., Imai Y., Niki E. (2009). Hydroxyoctadecadienoic acid and oxidatively modified peroxiredoxins in the blood of Alzheimer’s disease patients and their potential as biomarkers. Neurobiol. Aging.

[38] Dietrich-Muszalska A., Olas B. (2009). Isoprostenes as indicators of oxidative stress in schizophrenia. World J. Biol. Psychiatry.

[39] Chocholoušková M., Jirásko R., Vrána D., Gatěk J., Melichar B., Holčapek M. (2019). Reversed phase UHPLC/ESI-MS determination of oxylipins in human plasma: a case study of female breast cancer. Anal. Bioanal. Chem..

[40] Kapadia R., Yi J.-H., Vemuganti R. (2008). Mechanisms of anti-inflammatory and neuroprotective actions of PPAR-gamma agonists. Front. Biosci..

[41] Goetzl E.J., Gorman R.R. (1978). Chemotactic and chemokinetic stimulation of human eosinophil and neutrophil polymorphonuclear leukocytes by 12-L-hydroxy-5,8,10-heptadecatrienoic acid (HHT). J. Immunol..

[42] Zhang L., Chen B., Zhang J., Li J., Yang Q., Zhong Q., Zhan S., Liu H., Cai C. (2017). Serum polyunsaturated fatty acid metabolites as useful tool for screening potential biomarker of colorectal cancer. Prostaglandins Leukot. Essent. Fat. Acids.

[43] Yuan H., Li M.-Y., Ma L.T., Hsin M.K.Y., Mok T.S.K., Underwood M.J., Chen G.G. (2010). 15-Lipoxygenases and its metabolites 15(S)-HETE and 13(S)-HODE in the development of non-small cell lung cancer. Thorax.

[44] Larré S., Tran N., Fan C., Hamadeh H., Champigneulles J., Azzouzi R., Cussenot O., Mangin P., Olivier J.L. (2008). PGE2 and LTB4 tissue levels in benign and cancerous prostates. Prostaglandins Other Lipid Mediat..

[45] Spickett C.M., Pitt A.R. (2015). Oxidative Lipidomics Coming of Age: Advances in Analysis of Oxidized Phospholipids in Physiology and Pathology. Antioxid. Redox Signal..

[46] Lundström S.L., Levänen B., Nording M., Klepczynska-Nyström A., Sköld M., Haeggström J.Z., Grunewald J., Svartengren M., Hammock B.D., Larsson B.-M. (2011). Asthmatics Exhibit Altered Oxylipin Profiles Compared to Healthy Individuals after Subway Air Exposure. PLoS ONE.

[47] Yang J., Schmelzer K., Georgi K., Hammock B.D. (2009). Quantitative Profiling Method for Oxylipin Metabolome by Liquid Chromatography Electrospray Ionization Tandem Mass Spectrometry. Anal. Chem..

[48] Colas R.A., Shinohara M., Dalli J., Chiang N., Serhan C.N. (2014). Identification and signature profiles for pro-resolving and inflammatory lipid mediators in human tissue. AJP Cell Physiol..

[49] Golovko M.Y., Murphy E.J. (2008). An improved LC-MS/MS procedure for brain prostanoid analysis using brain fixation with head-focused microwave irradiation and liquid-liquid extraction. J. Lipid Res..

[50] Willenberg I., Ostermann A.I., Schebb N.H. (2015). Targeted metabolomics of the arachidonic acid cascade: current state and challenges of LC-MS analysis of oxylipins. Anal. Bioanal. Chem..

[51] Strassburg K., Huijbrechts A.M.L., Kortekaas K.A., Lindeman J.H., Pedersen T.L., Dane A., Berger R., Brenkman A., Hankemeier T., Van Duynhoven J. (2012). Quantitative profiling of oxylipins through comprehensive LC-MS/MS analysis: Application in cardiac surgery. Anal. Bioanal. Chem..

[52] Brose S.A., Baker A.G., Golovko M.Y. (2013). A Fast One-Step Extraction and UPLC–MS/MS Analysis for E2/D2 Series Prostaglandins and Isoprostanes. Lipids.

[53] Yuan Z.-X., Majchrzak-Hong S., Keyes G.S., Iadarola M.J., Mannes A.J., Ramsden C.E. (2018). Lipidomic profiling of targeted oxylipins with ultra-performance liquid chromatography-tandem mass spectrometry. Anal. Bioanal. Chem..

[54] Drake S.K., Bowen R.A.R., Remaley A.T., Hortin G.L. (2004). Potential Interferences from Blood Collection Tubes in Mass Spectrometric Analyses of Serum Polypeptides. Clin. Chem..

[55] Ito R., Miura N., Iguchi H., Nakamura H., Ushiro M., Wakui N., Nakahashi K., Iwasaki Y., Saito K., Suzuki T. (2008). Determination of tris(2-ethylhexyl)trimellitate released from PVC tube by LC–MS/MS. Int. J. Pharm..

[56] Schauer K.L., Broccardo C.J., Webb K.M., Covey P.A., Prenni J.E. (2013). Mass Spectrometry Contamination from Tinuvin 770, a Common Additive in Laboratory Plastics. J. Biomol. Tech..

[57] Haned Z., Moulay S., Lacorte S. (2018). Migration of plasticizers from poly(vinyl chloride) and multilayer infusion bags using selective extraction and GC–MS. J. Pharm. Biomed. Anal..

[58] Wang D., DuBois R.N. (2007). Measurement of Eicosanoids in Cancer Tissues. Methods Enzymol..

[59] Rund K.M., Ostermann A.I., Kutzner L., Galano J.M., Oger C., Vigor C., Wecklein S., Seiwert N., Durand T., Schebb N.H. (2018). Development of an LC-ESI(-)-MS/MS method for the simultaneous quantification of 35 isoprostanes and isofurans derived from the major n3- and n6-PUFAs. Anal. Chim. Acta.

[60] Fulton D., Falck J.R., McGiff J.C., Carroll M.A., Quilley J. (1998). A method for the determination of 5,6-EET using the lactone as an intermediate in the formation of the diol. J. Lipid Res..

[61] Araujo P., Mengesha Z., Lucena E., Grung B. (2014). Development and validation of an extraction method for the determination of pro-inflammatory eicosanoids in human plasma using liquid chromatography-tandem mass spectrometry. J. Chromatogr. A.

[62] Mueller M.J., Mène-Saffrané L., Grun C., Karg K., Farmer E.E. (2006). Oxylipin analysis methods. Plant J..

[63] Balvers M.G.J., Verhoeckx K.C.M., Bijlsma S., Rubingh C.M., Meijerink J., Wortelboer H.M., Witkamp R.F. (2012). Fish oil and inflammatory status alter the n-3 to n-6 balance of the endocannabinoid and oxylipin metabolomes in mouse plasma and tissues. Metabolomics.

[64] Hennebelle M., Otoki Y., Yang J., Hammock B.D., Levitt A.J., Taha A.Y., Swardfager W. (2017). Altered soluble epoxide hydrolase-derived oxylipins in patients with seasonal major depression: An exploratory study. Psychiatry Res..

[65] Hewawasam E., Liu G., Jeffery D.W., Muhlhausler B.S., Gibson R.A. (2018). A stable method for routine analysis of oxylipins from dried blood spots using ultra-high performance liquid chromatography–tandem mass spectrometry. Prostaglandins Leukot. Essent. Fat. Acids.

[66] Yang P., Felix E., Madden T., Fischer S.M., Newman R.A. (2002). Quantitative high-performance liquid chromatography/electrospray ionization tandem mass spectrometric analysis of 2- and 3-series prostaglandins in cultured tumor cells. Anal. Biochem..

[67] Newman J.W., Watanabe T., Hammock B.D. (2002). The simultaneous quantification of cytochrome P450 dependent linoleate and arachidonate metabolites in urine by HPLC-MS/MS. J. Lipid Res..

[68] Morgan A.H., Hammond V.J., Morgan L., Thomas C.P., Tallman K.A., Garcia-Diaz Y.R., McGuigan C., Serpi M., Porter N.A., Murphy R.C. (2010). Quantitative assays for esterified oxylipins generated by immune cells. Nat. Protoc..

[69] Dumlao D.S., Buczynski M.W., Norris P.C., Harkewicz R., Dennis E.A. (2011). High-throughput lipidomic analysis of fatty acid derived eicosanoids and N-acylethanolamines. Biochim. Biophys. Acta - Mol. Cell Biol. Lipids.

[70] Mesaros C., Lee S.H., Blair I.A. (2010). Analysis of epoxyeicosatrienoic acids by chiral liquid chromatography/electron capture atmospheric pressure chemical ionization mass spectrometry using [13C]-analog internal standards. Rapid Commun. Mass Spectrom..

[71] Lee Y.H., Cui L., Fang J., Chern B.S.M., Tan H.H., Chan J.K.Y. (2016). Limited value of pro-inflammatory oxylipins and cytokines as circulating biomarkers in endometriosis - A targeted ’omics study. Sci. Rep..

[72] Wang Y., Armando A.M., Quehenberger O., Yan C., Dennis E.A. (2014). Comprehensive ultra-performance liquid chromatographic separation and mass spectrometric analysis of eicosanoid metabolites in human samples. J. Chromatogr. A.

[73] Shearer G.C., Harris W.S., Pedersen T.L., Newman J.W. (2010). Detection of omega-3 oxylipins in human plasma and response to treatment with omega-3 acid ethyl esters. J. Lipid Res..

[74] Gouveia-Figueira S., Späth J., Zivkovic A.M., Nording M.L. (2015). Profiling the oxylipin and endocannabinoid metabolome by UPLC-ESI-MS/MS in human plasma to monitor postprandial inflammation. PLoS ONE.

[75] Gouveia-Figueira S., Nording M.L. (2015). Validation of a tandem mass spectrometry method using combined extraction of 37 oxylipins and 14 endocannabinoid-related compounds including prostamides from biological matrices. Prostaglandins Other Lipid Mediat..

[76] Schuchardt J.P., Schmidt S., Kressel G., Dong H., Willenberg I., Hammock B.D., Hahn A., Schebb N.H. (2013). Comparison of free serum oxylipin concentrations in hyper- vs. normolipidemic men. Prostaglandins Leukot. Essent. Fat. Acids.

[77] Hellström F., Gouveia-Figueira S., Nording M.L., Björklund M., Fowler C.J. (2016). Association between plasma concentrations of linoleic acid-derived oxylipins and the perceived pain scores in an exploratory study in women with chronic neck pain. BMC Musculoskelet. Disord..

[78] Astarita G., Kendall A.C., Dennis E.A., Nicolaou A. (2015). Targeted lipidomic strategies for oxygenated metabolites of polyunsaturated fatty acids. Biochim. Biophys. Acta - Mol. Cell Biol. Lipids.

[79] Maskrey B.H., O’Donnell V.B. (2008). Analysis of eicosanoids and related lipid mediators using mass spectrometry. Biochem. Soc. Trans..

[80] Tsikas D., Zoerner A.A. (2014). Analysis of eicosanoids by LC-MS/MS and GC-MS/MS: A historical retrospect and a discussion. J. Chromatogr. B Anal. Technol. Biomed. Life Sci..

[81] Schweer H., Kammer J., Kühl P.G., Seyberth H.W. (1986). Determination of peripheral plasma prostanoid concentration: an unreliable index of “in vivo” prostanoid activity. Eur. J. Clin. Pharmacol..

[82] Vuckovic D. (2012). Current trends and challenges in sample preparation for global metabolomics using liquid chromatography-mass spectrometry. Anal. Bioanal. Chem..

[83] Tan Z.-R., Ouyang D.-S., Zhou G., Wang L.-S., Li Z., Wang D., Zhou H.-H. (2006). Sensitive bioassay for the simultaneous determination of pseudoephedrine and cetirizine in human plasma by liquid-chromatography–ion trap spectrometry. J. Pharm. Biomed. Anal..

[84] Satomi Y., Hirayama M., Kobayashi H. (2017). One-step lipid extraction for plasma lipidomics analysis by liquid chromatography mass spectrometry. J. Chromatogr. B.

[85] Martin-Venegas R., Jáuregui O., Moreno J.J. (2014). Liquid chromatography-tandem mass spectrometry analysis of eicosanoids and related compounds in cell models. J. Chromatogr. B.

[86] Heemskerk M.M., Dharuri H.K., van den Berg S.A.A., Jónasdóttir H.S., Kloos D.-P., Giera M., van Dijk K.W., van Harmelen V. (2014). Prolonged niacin treatment leads to increased adipose tissue PUFA synthesis and anti-inflammatory lipid and oxylipin plasma profile. J. Lipid Res..

[87] Polson C., Sarkar P., Incledon B., Raguvaran V., Grant R. (2003). Optimization of protein precipitation based upon effectiveness of protein removal and ionization effect in liquid chromatography-tandem mass spectrometry. J. Chromatogr. B. Analyt. Technol. Biomed. Life Sci..

[88] Zein Elabdeen H.R., Mustafa M., Szklenar M., Rühl R., Ali R., Bolstad A.I. (2013). Ratio of Pro-Resolving and Pro-Inflammatory Lipid Mediator Precursors as Potential Markers for Aggressive Periodontitis. PLoS ONE.

[89] Wang W., Qin S., Li L., Chen X., Wang Q., Wei J. (2015). An Optimized High Throughput Clean-Up Method Using Mixed-Mode SPE Plate for the Analysis of Free Arachidonic Acid in Plasma by LC-MS/MS. Int. J. Anal. Chem..

[90] Kortz L., Helmschrodt C., Ceglarek U. (2011). Fast liquid chromatography combined with mass spectrometry for the analysis of metabolites and proteins in human body fluids. Anal. Bioanal. Chem..

[91] Kortz L., Dorow J., Becker S., Thiery J., Ceglarek U. (2013). Fast liquid chromatography-quadrupole linear ion trap-mass spectrometry analysis of polyunsaturated fatty acids and eicosanoids in human plasma. J. Chromatogr. B Anal. Technol. Biomed. Life Sci..

[92] Klawitter J., Haschke M., Shokati T., Klawitter J., Christians U. (2011). Quantification of 15-F2t-isoprostane in human plasma and urine: results from enzyme-linked immunoassay and liquid chromatography/tandem mass spectrometry cannot be compared. Rapid Commun. Mass Spectrom..

[93] Bessonneau V., Zhan Y., De Lannoy I.A.M., Saldivia V., Pawliszyn J. (2015). In vivo solid-phase microextraction liquid chromatography-tandem mass spectrometry for monitoring blood eicosanoids time profile after lipopolysaccharide-induced inflammation in Sprague-Dawley rats. J. Chromatogr. A.

[94] Tumanov S., Kamphorst J.J. (2017). Recent advances in expanding the coverage of the lipidome. Curr. Opin. Biotechnol..

[95] Folch J., Lees M., Sloane Stanley G.H. (1957). A simple method for the isolation and purification of total lipides from animal tissues. J. Biol. Chem..

[96] Bligh E.G., Dyer W.J. (1959). A Rapid Method of Total Lipid Extraction and Purification. Can. J. Physiol. Pharmacol..

[97] Puppolo M., Varma D., Jansen S.A. (2014). A review of analytical methods for eicosanoids in brain tissue. J. Chromatogr. B Anal. Technol. Biomed. Life Sci..

[98] Yang Y., Cruickshank C., Armstrong M., Mahaffey S., Reisdorph R., Reisdorph N. (2013). New sample preparation approach for mass spectrometry-based profiling of plasma results in improved coverage of metabolome. J. Chromatogr. A.

[99] Fromel T., Jungblut B., Hu J., Trouvain C., Barbosa-Sicard E., Popp R., Liebner S., Dimmeler S., Hammock B.D., Fleming I. (2012). Soluble epoxide hydrolase regulates hematopoietic progenitor cell function via generation of fatty acid diols. Proc. Natl. Acad. Sci..

[100] Pier B., Edmonds J.W., Wilson L., Arabshahi A., Moore R., Bates G.W., Prasain J.K., Miller M.A. (2018). Comprehensive profiling of prostaglandins in human ovarian follicular fluid using mass spectrometry. Prostaglandins Other Lipid Mediat..

[101] Larose J., Julien P., Bilodeau J.-F. (2013). Analysis of F 2 -isoprostanes in plasma of pregnant women by HPLC-MS/MS using a column packed with core-shell particles. J. Lipid Res..

[102] Hall L.M., Murphy R.C. (1998). Electrospray mass spectrometric analysis of 5-hydroperoxy and 5-hydroxyeicosatetraenoic acids generated by lipid peroxidation of red blood cell ghost phospholipids. J. Am. Soc. Mass Spectrom..

[103] Brose S.A., Thuen B.T., Golovko M.Y. (2011). LC/MS/MS method for analysis of E 2 series prostaglandins and isoprostanes. J. Lipid Res..

[104] Urban M., Enot D.P., Dallmann G., Körner L., Forcher V., Enoh P., Koal T., Keller M., Deigner H.P. (2010). Complexity and pitfalls of mass spectrometry-based targeted metabolomics in brain research. Anal. Biochem..

[105] Kempen E.C., Yang P., Felix E., Madden T., Newman R.A. (2001). Simultaneous Quantification of Arachidonic Acid Metabolites in Cultured Tumor Cells Using High-Performance Liquid Chromatography/Electrospray Ionization Tandem Mass Spectrometry. Anal. Biochem..

[106] Schroeder C.P., Yang P., Newman R.A., Lotan R. (2004). Eicosanoid metabolism in squamous cell carcinoma cell lines derived from primary and metastatic head and neck cancer and its modulation by celecoxib. Cancer Biol. Ther..

[107] Michaelis U.R., Xia N., Barbosa-Sicard E., Falck J.R., Fleming I. (2008). Role of cytochrome P450 2C epoxygenases in hypoxia-induced cell migration and angiogenesis in retinal endothelial cells. Investig. Ophthalmol. Vis. Sci..

[108] Cao H., Xiao L., Park G., Wang X., Azim A.C., Christman J.W., van Breemen R.B. (2008). An improved LC–MS/MS method for the quantification of prostaglandins E2 and D2 production in biological fluids. Anal. Biochem..

[109] (1998). Guide to Solid Phase Extraction. SUPELCO Bull. 910.

[110] Späth J. (2014). Oxylipins in human plasma – method development and dietary effects on levels. Master’s Thesis.

[111] VanRollins M., VanderNoot V.A. (2003). Simultaneous resolution of underivatized regioisomers and stereoisomers of arachidonate epoxides by capillary electrophoresis. Anal. Biochem..

[112] Ostermann A.I., Willenberg I., Schebb N.H. (2015). Comparison of sample preparation methods for the quantitative analysis of eicosanoids and other oxylipins in plasma by means of LC-MS/MS. Anal. Bioanal. Chem..

[113] Masoodi M., Mir A.A., Petasis N.A., Serhan C.N., Nicolaou A. (2008). Simultaneous lipidomic analysis of three families of bioactive lipid mediators leukotrienes, resolvins, protectins and related hydroxy-fatty acids by liquid chromatography/electrospray ionisation tandem mass spectrometry. Rapid Commun. Mass Spectrom..

[114] Galvão A.F., Petta T., Flamand N., Bollela V.R., Silva C.L., Jarduli L.R., Malmegrim K.C.R., Simões B.P., de Moraes L.A.B., Faccioli L.H. (2016). Plasma eicosanoid profiles determined by high-performance liquid chromatography coupled with tandem mass spectrometry in stimulated peripheral blood from healthy individuals and sickle cell anemia patients in treatment. Anal. Bioanal. Chem..

[115] Okemoto K., Maekawa K., Tajima Y., Tohkin M., Saito Y. (2016). Cross-Classification of Human Urinary Lipidome by Sex, Age, and Body Mass Index. PLoS ONE.

[116] Balgoma D., Larsson J., Rokach J., Lawson J.A., Daham K., Dahlén B., Dahlén S.-E., Wheelock C.E. (2013). Quantification of Lipid Mediator Metabolites in Human Urine from Asthma Patients by Electrospray Ionization Mass Spectrometry: Controlling Matrix Effects. Anal. Chem..

[117] Sterz K., Scherer G., Ecker J. (2012). A simple and robust UPLC-SRM/MS method to quantify urinary eicosanoids. J. Lipid Res..

[118] Medina S., Domínguez-Perles R., Gil J.I., Ferreres F., García-Viguera C., Martínez-Sanz J.M., Gil-Izquierdo A. (2012). A ultra-pressure liquid chromatography/triple quadrupole tandem mass spectrometry method for the analysis of 13 eicosanoids in human urine and quantitative 24 hour values in healthy volunteers in a controlled constant diet. Rapid Commun. Mass Spectrom..

[119] Gouveia-Figueira S., Karimpour M., Bosson J.A., Blomberg A., Unosson J., Pourazar J., Sandström T., Behndig A.F., Nording M.L. (2017). Mass spectrometry profiling of oxylipins, endocannabinoids, and N-acylethanolamines in human lung lavage fluids reveals responsiveness of prostaglandin E2 and associated lipid metabolites to biodiesel exhaust exposure. Anal. Bioanal. Chem..

[120] Panthi S., Chen J., Wilson L., Nichols J.J. (2018). Detection of Lipid Mediators of Inflammation in the Human Tear Film. Eye Contact Lens Sci. Clin. Pract..

[121] Giera M., Ioan-Facsinay A., Toes R., Gao F., Dalli J., Deelder A.M., Serhan C.N., Mayboroda O.A. (2012). Lipid and lipid mediator profiling of human synovial fluid in rheumatoid arthritis patients by means of LC–MS/MS. Biochim. Biophys. Acta - Mol. Cell Biol. Lipids.

[122] Rauzi F., Kirkby N.S., Edin M.L., Whiteford J., Zeldin D.C., Mitchell J.A., Warner T.D. (2016). Aspirin inhibits the production of proangiogenic 15( S )-HETE by platelet cyclooxygenase-1. FASEB J..

[123] Wang C., Colas R.A., Dalli J., Arnardottir H.H., Nguyen D., Hasturk H., Chiang N., Van Dyke T.E., Serhan C.N. (2016). Maresin 1 Biosynthesis and Proresolving Anti-infective Functions with Human-Localized Aggressive Periodontitis Leukocytes. Infect. Immun..

[124] Wu J., Gouveia-Figueira S., Domellöf M., Zivkovic A.M., Nording M.L. (2016). Oxylipins, endocannabinoids, and related compounds in human milk: Levels and effects of storage conditions. Prostaglandins Other Lipid Mediat..

[125] Robinson D.T., Palac H.L., Baillif V., Van Goethem E., Dubourdeau M., Van Horn L., Martin C.R. (2017). Long chain fatty acids and related pro-inflammatory, specialized pro-resolving lipid mediators and their intermediates in preterm human milk during the first month of lactation. Prostaglandins Leukot. Essent. Fat. Acids.

[126] Yue H., Strauss K.I., Borenstein M.R., Barbe M.F., Rossi L.J., Jansen S.A. (2004). Determination of bioactive eicosanoids in brain tissue by a sensitive reversed-phase liquid chromatographic method with fluorescence detection. J. Chromatogr. B Anal. Technol. Biomed. Life Sci..

[127] O’Donnell V.B., Maskrey B., Taylor G.W. (2009). Eicosanoids: Generation and detection in mammalian cells. Methods Mol Biol.

[128] Blewett A.J., Varma D., Gilles T., Libonati J.R., Jansen S.A. (2008). Development and validation of a high-performance liquid chromatography-electrospray mass spectrometry method for the simultaneous determination of 23 eicosanoids. J. Pharm. Biomed. Anal..

[129] Le Faouder P., Baillif V., Spreadbury I., Motta J.-P., Rousset P., Chêne G., Guigné C., Tercé F., Vanner S., Vergnolle N. (2013). LC–MS/MS method for rapid and concomitant quantification of pro-inflammatory and pro-resolving polyunsaturated fatty acid metabolites. J. Chromatogr. B.

[130] Weylandt K.H., Krause L.F., Gomolka B., Chiu C.Y., Bilal S., Nadolny A., Waechter S.F., Fischer A., Rothe M., Kang J.X. (2011). Suppressed liver tumorigenesis in fat-1 mice with elevated omega-3 fatty acids is associated with increased omega-3 derived lipid mediators and reduced TNF-α. Carcinogenesis.

[131] Jelińska M., Białek A., Mojska H., Gielecińska I., Tokarz A. (2014). Effect of conjugated linoleic acid mixture supplemented daily after carcinogen application on linoleic and arachidonic acid metabolites in rat serum and induced tumours. Biochim. Biophys. Acta - Mol. Basis Dis..

[132] Deems R., Buczynski M.W., Bowers-Gentry R., Harkewicz R., Dennis E.A. (2007). Detection and Quantitation of Eicosanoids via High Performance Liquid Chromatography-Electrospray Ionization-Mass Spectrometry. Methods in Enzymology.

[133] Takabatake M., Hishinuma T., Suzuki N., Chiba S., Tsukamoto H., Nakamura H., Saga T., Tomioka Y., Kurose A., Sawai T. (2002). Simultaneous quantification of prostaglandins in human synovial cell-cultured medium using liquid chromatography/tandem mass spectrometry. Prostaglandins Leukot. Essent. Fat. Acids.

[134] Masoodi M., Nicolaou A. (2006). Lipidomic analysis of twenty-seven prostanoids and isoprostanes by liquid chromatography/electrospray tandem mass spectrometry. Rapid Commun. Mass Spectrom..

[135] Tajima Y., Ishikawa M., Maekawa K., Murayama M., Senoo Y., Nishimaki-Mogami T., Nakanishi H., Ikeda K., Arita M., Taguchi R. (2013). Lipidomic analysis of brain tissues and plasma in a mouse model expressing mutated human amyloid precursor protein/tau for Alzheimer’s disease. Lipids Health Dis..

[136] Rago B., Fu C. (2013). Development of a high-throughput ultra performance liquid chromatography–mass spectrometry assay to profile 18 eicosanoids as exploratory biomarkers for atherosclerotic diseases. J. Chromatogr. B.

[137] Margalit A., Duffin K.L., Isakson P.C. (1996). Rapid Quantitation of a Large Scope of Eicosanoids in Two Models of Inflammation: Development of an Electrospray and Tandem Mass Spectrometry Method and Application to Biological Studies. Anal. Biochem..

[138] Dalli J., Serhan C.N. (2012). Specific lipid mediator signatures of human phagocytes: microparticles stimulate macrophage efferocytosis and pro-resolving mediators. Blood.

[139] Sanaki T., Fujihara T., Iwamoto R., Yoshioka T., Higashino K., Nakano T., Numata Y. (2015). Improvements in the High-Performance Liquid Chromatography and Extraction Conditions for the Analysis of Oxidized Fatty Acids Using a Mixed-Mode Spin Column. Mod. Chem. Appl..

[140] Petta T., Moraes L.A.B., Faccioli L.H. (2015). Versatility of tandem mass spectrometry for focused analysis of oxylipids. J. Mass Spectrom..

[141] Shinde D.D., Kim K.-B., Oh K.-S., Abdalla N., Liu K.-H., Bae S.K., Shon J.-H., Kim H.-S., Kim D.-H., Shin J.G. (2012). LC–MS/MS for the simultaneous analysis of arachidonic acid and 32 related metabolites in human plasma: Basal plasma concentrations and aspirin-induced changes of eicosanoids. J. Chromatogr. B.

[142] Chen G., Zhang Q. (2019). Comprehensive analysis of oxylipins in human plasma using reversed-phase liquid chromatography-triple quadrupole mass spectrometry with heatmap-assisted selection of transitions. Anal. Bioanal. Chem..

[143] Yasumoto A., Tokuoka S.M., Kita Y., Shimizu T., Yatomi Y. (2017). Multiplex quantitative analysis of eicosanoid mediators in human plasma and serum: Possible introduction into clinical testing. J. Chromatogr. B.

[144] Thakare R., Chhonker Y.S., Gautam N., Nelson A., Casaburi R., Criner G., Dransfield M.T., Make B., Schmid K.K., Rennard S.I. (2018). Simultaneous LC-MS/MS analysis of eicosanoids and related metabolites in human serum, sputum and BALF. Biomed. Chromatogr..

[145] Miller T.M., Donnelly M.K., Crago E.A., Roman D.M., Sherwood P.R., Horowitz M.B., Poloyac S.M. (2009). Rapid, simultaneous quantitation of mono and dioxygenated metabolites of arachidonic acid in human CSF and rat brain. J. Chromatogr. B Anal. Technol. Biomed. Life Sci..

[146] Bollinger J.G., Thompson W., Lai Y., Oslund R.C., Hallstrand T.S., Sadilek M., Turecek F., Gelb M.H. (2010). Improved Sensitivity Mass Spectrometric Detection of Eicosanoids by Charge Reversal Derivatization. Anal. Chem..

[147] Lee C.-Y.J., Jenner A., Halliwell B. (2004). Rapid preparation of human urine and plasma samples for analysis of F2-isoprostanes by gas chromatography-mass spectrometry. Biochem. Biophys. Res. Commun..

[148] Caligiuri S.P.B., Aukema H.M., Ravandi A., Guzman R., Dibrov E., Pierce G.N. (2014). Flaxseed consumption reduces blood pressure in patients with hypertension by altering circulating oxylipins via an α-linolenic acid-induced inhibition of soluble epoxide hydrolase. Hypertension.

[149] Song J., Liu X., Wu J., Meehan M.J., Blevitt J.M., Dorrestein P.C., Milla M.E. (2013). A highly efficient, high-throughput lipidomics platform for the quantitative detection of eicosanoids in human whole blood. Anal. Biochem..

[150] Lebold K.M., Kirkwood J.S., Taylor A.W., Choi J., Barton C.L., Miller G.W., La Du J., Jump D.B., Stevens J.F., Tanguay R.L. (2014). Novel liquid chromatography–mass spectrometry method shows that vitamin E deficiency depletes arachidonic and docosahexaenoic acids in zebrafish (Danio rerio) embryos. Redox Biol..

[151] García-Flores L.A., Medina S., Gómez C., Wheelock C.E., Cejuela R., Martínez-Sanz J.M., Oger C., Galano J.M., Durand T., Hernández-Sáez Á. (2018). Aronia - Citrus juice (polyphenol-rich juice) intake and elite triathlon training: A lipidomic approach using representative oxylipins in urine. Food Funct..

[152] Mizuno K., Kataoka H. (2015). Analysis of urinary 8-isoprostane as an oxidative stress biomarker by stable isotope dilution using automated online in-tube solid-phase microextraction coupled with liquid chromatography-tandem mass spectrometry. J. Pharm. Biomed. Anal..

[153] Rodríguez Patiño G., Castillo Rodríguez M.A., Ramírez Bribiesca J.E., Ramírez Noguera P., Gonsebatt Bonaparte M.E., López-Arellano R. (2018). Development of a method for the determination of 8-iso-PGF2α in sheep and goat plasma using solid-phase microextraction and ultra-performance liquid chromatography/tandem mass spectrometry. Rapid Commun. Mass Spectrom..

[154] Hyötyläinen T. (2009). Critical evaluation of sample pretreatment techniques. Anal. Bioanal. Chem..

[155] Prosen H. (2014). Applications of liquid-phase microextraction in the sample preparation of environmental solid samples. Molecules.

[156] Perestrelo R., Silva C.L., Câmara J.S. (2015). Determination of urinary levels of leukotriene B4 using ad highly specific and sensitive methodology based on automatic MEPS combined with UHPLC-PDA analysis. Talanta.

[157] Suhr A.C., Bruegel M., Maier B., Holdt L.M., Kleinhempel A., Teupser D., Grimm S.H., Vogeser M. (2016). Ferromagnetic particles as a rapid and robust sample preparation for the absolute quantification of seven eicosanoids in human plasma by UHPLC-MS/MS. J. Chromatogr. B Anal. Technol. Biomed. Life Sci..

[158] Balashova E.E., Trifonova O.P., Maslov D.L., Lokhov P.G. (2018). Application of dried blood spot for analysis of low molecular weight fraction ( metabolome ) of blood. Heal. Prim. Care.

[159] Ferreiro-Vera C., Mata-Granados J.M., Priego-Capote F., Quesada-Gómez J.M., Luque de Castro M.D. (2011). Automated targeting analysis of eicosanoid inflammation biomarkers in human serum and in the exometabolome of stem cells by SPE–LC–MS/MS. Anal. Bioanal. Chem..

[160] Wagner B.M. (2014). Entwicklung Eines Multidimensionalen bioanalytischen Modellsystems für die Gesicherte Identifikation der Auswirkungen von Oxidativen Stressfaktoren auf den Organismus. Ph.D. Thesis.

[161] Kita Y., Takahashi T., Uozumi N., Shimizu T. (2005). A multiplex quantitation method for eicosanoids and platelet-activating factor using column-switching reversed-phase liquid chromatography–tandem mass spectrometry. Anal. Biochem..

[162] Parkinson D.R. (2012). Analytical Derivatization Techniques. Comprehensive Sampling and Sample Preparation.

[163] Moraes L.A., Giner R.M., Paul-Clark M.J., Perretti M., Perrett D. (2004). An isocratic HPLC method for the quantitation of eicosanoids in human platelets. Biomed. Chromatogr..

[164] Nithipatikom K., Pratt P.F., Campbell W.B. (2000). Determination of EETs using microbore liquid chromatography with fluorescence detection. Am. J. Physiol. Circ. Physiol..

[165] Chavis C., Fraissinet L., Chanez P., Thomas E., Bousquet J. (1999). A Method for the Measurement of Plasma Hydroxyeicosatetraenoic Acid Levels. Anal. Biochem..

[166] Aghazadeh-Habashi A., Asghar W., Jamali F. (2015). Simultaneous determination of selected eicosanoids by reversed-phase HPLC method using fluorescence detection and application to rat and human plasma, and rat heart and kidney samples. J. Pharm. Biomed. Anal..

[167] Meckelmann S.W., Hellhake S., Steuck M., Krohn M., Schebb N.H. (2017). Comparison of derivatization/ionization techniques for liquid chromatography tandem mass spectrometry analysis of oxylipins. Prostaglandins Other Lipid Mediat..

[168] Schulze B. (2005). Oxylipins and their involvement in plant response to biotic and abiotic stress. Ph.D. Thesis.

[169] Mesaros C., Blair I.A. (2012). Targeted Chiral Analysis of Bioactive Arachidonic Acid Metabolites Using Liquid-Chromatography-Mass Spectrometry. Metabolites.

[170] Knott I., Dieu M., Burton M., Lecomte V., Remacle J., Raes M. (1993). Differential effects of interleukin-1α and β on the arachidonic acid cascade in human synovial cells and chondrocytes in culture. Agents Actions.

[171] Jira W., Spitellera G., Richter A. (1998). Increased Levels of Lipid Oxidation Products in Rheumatically Destructed Bones of Patients Suffering from Rheumatoid Arthritis. Zeitschrift Naturforsch. Sect. C J. Biosci..

[172] Molnár-Perl I., Wilson I., Poole C., Cooke M. (2000). AMINO ACIDS | Liquid Chromatography. Encyclopedia of Separation Science.

[173] Prinsen H.C.M.T., Schiebergen-Bronkhorst B.G.M., Roeleveld M.W., Jans J.J.M., de Sain-van der Velden M.G.M., Visser G., van Hasselt P.M., Verhoeven-Duif N.M. (2016). Rapid quantification of underivatized amino acids in plasma by hydrophilic interaction liquid chromatography (HILIC) coupled with tandem mass-spectrometry. J. Inherit. Metab. Dis..

[174] Quehenberger O., Armando A.M., Brown A.H., Milne S.B., Myers D.S., Merrill A.H., Bandyopadhyay S., Jones K.N., Kelly S., Shaner R.L. (2010). Lipidomics reveals a remarkable diversity of lipids in human plasma. J. Lipid Res..

[175] VanderNoot V.A., VanRollins M. (2002). Capillary Electrophoresis of Cytochrome P-450 Epoxygenase Metabolites of Arachidonic Acid. 2. Resolution of Stereoisomers. Anal. Chem..

